# Socioeconomic value of adult respiratory vaccination in the United States: a benefit-cost analysis

**DOI:** 10.1093/haschl/qxag114

**Published:** 2026-05-12

**Authors:** Claud Theakston, Tianyan Hu, Simon Brassel, Jeffrey Vietri, Diana Mendes, Matthew Napier, Ellie Tunnicliffe, Alon Yehoshua, Raymond Farkouh, Lotte Steuten

**Affiliations:** Office of Health Economics, London SE1 2HB, United Kingdom; Pfizer Inc., NewYork, NY 10001, United States; Office of Health Economics, London SE1 2HB, United Kingdom; Pfizer Inc., Collegeville, PA 19426, United States; Pfizer Ltd., Tadworth, Surrey KT20 7NS, United Kingdom; Office of Health Economics, London SE1 2HB, United Kingdom; Office of Health Economics, London SE1 2HB, United Kingdom; Pfizer Inc., NewYork, NY 10001, United States; Pfizer Inc., NewYork, NY 10001, United States; Office of Health Economics, London SE1 2HB, United Kingdom

**Keywords:** benefit-cost analysis, BCA, economic evaluation, health policy, vaccines, adult respiratory vaccination

## Abstract

**Background:**

Respiratory infections, including pneumococcal disease (PD), respiratory syncytial virus (RSV), influenza, and COVID-19, impose substantial health and economic burdens on adults in the United States. Although vaccines are available, coverage remains suboptimal.

**Methods:**

We conducted a societal-perspective benefit–cost analysis of adult PD, RSV, influenza, and COVID-19 vaccination programs. Four disease models estimated averted morbidity, mortality, and productivity losses. We evaluated current CDC recommendations and scenarios of varying eligibility and coverage. A complementary analysis estimated the net present value of 1 year's vaccination activity.

**Results:**

Under current age-based recommendations, vaccination prevents over 182 000 deaths and 2.5 million hospitalizations within 15 years. Society values these outcomes at 5-12 times the cost of delivering the programs. Including at-risk adults averts an additional 13 600 deaths. Restricting eligibility or coverage could increase vaccine-preventable hospitalizations by over 1 million, increasing healthcare costs by almost $50 billion; expanding eligibility and increasing coverage to 75% substantially improves societal welfare and could additionally avert over 100 000 deaths. One year's vaccination activity prevents ∼300 000 hospitalizations, and ∼18 500 deaths, generating $6–$12 in societal value per $1 spent.

**Conclusions:**

Adult respiratory vaccination programs generate considerable societal returns. Increasing coverage and sustaining clear, evidence-based recommendations can unlock further health and economic gains.

## Introduction

Respiratory infections impose a substantial burden on adult health, healthcare systems, and the economy in the United States (US). Four vaccine-preventable respiratory diseases in particular—pneumococcal disease (PD), respiratory syncytial virus (RSV) associated disease, influenza, and COVID-19—are each associated with 10 000–50 000 annual fatalities,^1-4^ and between 100 000 and almost half a million annual hospitalizations.^5-7^ However, due to low testing rates and associated data and methodological limitations, the true burden is likely much higher.^8-11^ Each of these respiratory diseases annually imposes direct costs to the healthcare system of up to $4 billion,^12-15^ as well as often much higher indirect socioeconomic costs, such as productivity loss, each costing up to tens of billions of dollars.^13,16-18^

Much of this burden is preventable through the use of safe and effective vaccines that are licensed, recommended, and accessible. Indeed, vaccination is the most effective and scalable strategy to reduce disease-associated mortality, morbidity and healthcare system pressure.^19-25^ The Advisory Committee on Immunization Practices (ACIP), a committee within the US Centers for Disease Control and Prevention (CDC), is responsible for issuing evidence-based recommendations that the CDC may adopt as guidance for routine vaccination. As part of that evaluation process, the economic efficiency, or value for money as demonstrated by favorable cost-effectiveness profiles from both payer and societal perspectives, is assessed.^26-28^ Among the adult population, current CDC guidance recommends routine vaccination against PD for adults 50+ and at-risk adults aged 18-49, RSV for adults 75+ and at-risk adults aged 50-74, influenza for all adults aged 18+, and COVID-19 for all adults 18+^29^ under shared clinical decision-making (SCDM), which emphasizes that the risk-benefit of vaccination is most favorable for those at increased risk of severe disease. The CDC and other societies, such as the Infectious Disease Society of America, emphasize that RSV, influenza and COVID vaccines can be co-administered safely together at one visit.^30,31^

Despite these recommendations, vaccination coverage in the US remains below international benchmarks. The World Health Organization (WHO) sets a 75% influenza coverage target for adults over 65,^32^ a target that several European nations approach or occasionally exceed, and increasingly extend this target to RSV and COVID-19.^33-43^ However, the highest coverage level achieved within the US is roughly 65%, specifically among adults over 65 protected against PD and influenza. Coverage in younger age groups, including those at elevated risk, or other disease areas, such as RSV and COVID-19, falls significantly behind.^44-46^ These low coverage rates limit the potential public health and socioeconomic welfare benefits these programs offer. This is further compounded by coverage disparities by age, race/ethnicity, and socioeconomic status,^26^ along with significant and enduring levels of vaccine hesitancy.^47^ Access and affordability are also influenced by policy. CDC recommendations determine which vaccines are covered without cost-sharing. Additionally, Medicaid disenrollments following the pandemic and the expiration of the federal Bridge Access Program have reduced coverage for millions of adults.^48-51^

Public spending and investment decisions should reflect societal preferences and aim to maximize societal welfare subject to available resources and policy constraints. Cost-effectiveness analyses (CEA), commonly used to inform vaccine policy, assess how best to allocate a limited healthcare budget but do not, in most cases, capture the full societal value of health interventions^52^ or allow comparison across policy domains.^53^ Benefit-cost analyses (BCA) complement CEA by monetizing health and non-health outcomes, providing evidence of the investment value of a policy or program that is comparable across governmental departments.^52-55^ While BCAs have been conducted in other domains,^56^ there is a lack of such studies assessing adult vaccination. Additionally, existing economic assessments of respiratory vaccination often focus on pediatric populations or single diseases.^57,58^ Consequently, decision-makers lack a comparable, economy-wide estimate of the full societal value and economic returns from adult respiratory vaccination programs that accounts for medical expenditure offsets, productivity effects and societal valuation of prevented mortality across multiple vaccines simultaneously.

To inform policy and budget decisions, this study estimates the socioeconomic benefits of current US adult vaccination programs for PD, RSV, influenza, and COVID-19 using a BCA from a societal-perspective. We also evaluate a set of scenarios varying eligibility and coverage to illustrate how policy and programmatic choices affect societal returns. By quantifying their full value, we aim to support evidence-based policies that sustain and expand the health and economic benefits of adult vaccination.

## Data and methods

Four disease models estimate morbidity and mortality reductions attributable to each vaccination program, following a previously published approach.^20,21^ The models are static, meaning they do not capture the complex transmission dynamics underlying the four diseases and therefore do not reflect the potential impact of indirect (herd) protection among unvaccinated individuals. They are designed as closed-cohort models, representing the current U.S. demographic structure without projecting future demographic changes. All models are multi-cohort, following each eligible age and/or risk cohort in annual model cycles from the time of vaccination through death, the end of the considered time horizon or age 100, whichever comes first.

All costs and outcomes are fed into an established BCA framework.^54^ Outcomes are aggregated within three categories: morbidity risk reduction, mortality risk reduction and averted work productivity loss.

The value of morbidity risk reduction is proxied through the medical costs associated with each illness. Mortality risk reduction is valued using two alternatives: in the first, the number of fatal events averted is multiplied by the value of a statistical life (VSL) provided by the US Department of Health and Human Services,^59^ assuming independence from age. Alternatively, averted life years lost is multiplied by the Value of a Statistical Life Year (VSLY), a derivative of the VSL, which lowers the value of mortality risk reduction for older ages. We report both VSL and VSLY to reflect alternative normative valuation choices relevant to policy. We estimate productivity losses as lost wages from work absences caused by respiratory illness. We perform robustness checks using human capital method estimates to value mortality and productivity losses, available in the Technical Appendix.

We calculate overall Benefit-Cost Ratios (BCR), return on investment (ROI), and Net Benefits (NB) over 1-, 7-, and 15-year horizons, reflecting the duration of protection and hence the maximum aggregable benefits from each program. Influenza and COVID-19 vaccines are recommended annually, while vaccines for RSV and PD are administered with a single-dose due to their longer estimated duration of protection, projected to wane to 0% by 7 and 15 years, respectively.^60,61^ Future costs and benefits are discounted at 2%.^62^ Our primary analysis captures the value of the full program/current recommendations over time. We also include an additional analysis in the Technical Appendix, which models the net present value of 1 year's vaccination activity. This encompasses vaccination of the full eligible population against influenza and COVID-19, as well as single-dose vaccination against PD and RSV for the newly eligible cohort, in line with current recommendations.

**Figure 1.**
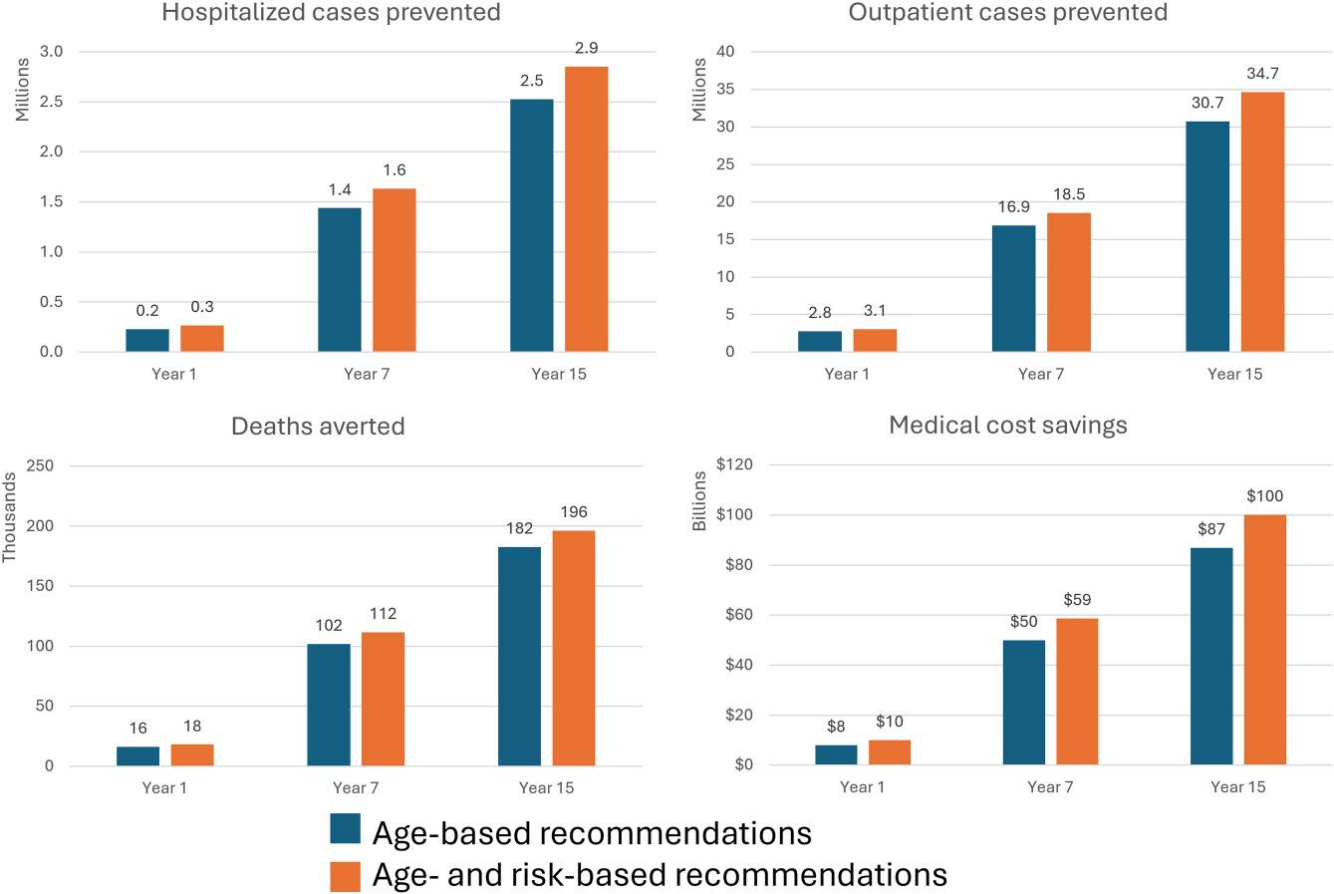
Healthcare resource impact and monetized benefits.

We quantify outcomes using recently observed coverage rates (Table 1) and report two sets of primary results: (1) current CDC age-based recommendations and (2) current CDC age-based and risk-based recommendations. For COVID-19, the base case focuses on the vaccine label indication,^66^ modeling vaccination of adults aged 65 and older and at-risk adults aged 18-64, reflecting the populations for whom the risk-benefit of vaccination is most favorable under the current SCDM recommendation.^67^ The potential impact of broader COVID-19 vaccination among all adults aged 18+ is examined in a scenario. We also report additional sub-outcomes, including hospitalizations averted, hospital bed-days averted, outpatient visits averted, and direct medical costs offset (Figure 1). All monetary values are presented in 2024 USD.

**Table 1.** Current program specifications and coverage rates.

	Age-based analytic set		Risk-based analytic set	
	Eligibility	Coverage	Eligibility	Coverage
PD^a^	50+	64%^63^	18-49	23%^26^
RSV^a^	75+	21%^64^	50-74	15%^64^
Influenza^b^	18+	33%-66%^46^	N/A	N/A
COVID-19^b^	65+	47%^65^	18-64	13%-23%^65^

^a^Administered as a single, one-off dose.

^b^Administered annually.

Additionally, we examined six policy-relevant scenarios, grouped into two categories: restrictive scenarios that reduce eligibility/coverage, and expansive scenarios that increase eligibility/coverage.

Restrictive scenarios (R1–R3)

**R1 – Restricted eligibility to ages ≥75:** Age-based recommendations for all four programs are limited to adults aged 75 years and older.
**R2 – Reduced coverage to 25%:** Coverage is capped at 25% in all programs for populations where observed coverage currently exceeds 25%; coverage levels already below 25% (eg, RSV and some at-risk groups) are left unchanged.
**R3 – Combined restriction:** Applies both R1 and R2 simultaneously.

Expansive scenarios (E1–E3)

**E1 – Expanded COVID-19 eligibility to ages ≥18:** COVID-19 age-based eligibility extended to all adults aged 18 years and older, reflecting the actual CDC SCDM recommendations.
**E2 – Increased coverage to 75%:** Coverage for all four programs is increased to 75%, consistent with international benchmarks.
**E3 – Combined expansion:** Applies both E1 and E2 simultaneously.

To reflect relative change to the status quo, each scenario is compared with the current CDC age- and risk-based recommendations. Results are compared at year 15, as after this time, the effectiveness of the program that offers the longest duration of protection (PD) has fully waned.

One-way sensitivity analyses test the robustness of results. Full results for all analyses, and descriptions of the models and parameter inputs are included in the Technical Appendix.

## Results

### Primary results

Over 15 years, the current CDC age-based recommendations are estimated to prevent over 2.5 million hospitalizations, freeing up 15 million hospital bed days, as well as preventing almost 31 million outpatient appointments, and preventing over 182 000 deaths (Figure 1). When valuing these using the VSLY, this corresponds to NBs of $746 billion, corresponding to a BCR of 5:1 (Table 2), meaning $5 in societal value is generated for every $1 spent on the four vaccination programs. When valued using the VSL, the respective NBs exceed $2.3 trillion, with a BCR of 12:1.

**Table 2.** Aggregated discounted BCRs, ROIs and NBs for the current age-based and age- and risk-based recommendations.

Age-based recommendations			
Time Horizon	Year 1	Year 7	Year 15
Costs	$45.70B	$126.00B	$204.52B
VSL			
Benefits	$225.67B	$1427.33B	$2555.80B
BCR	5	11	12
ROI	394%	1033%	1150%
NB	$179.97B	$1301.33B	$2351.28B
VSLY			
Benefits	$87.29B	$543.54B	$950.26B
BCR	2	4	5
ROI	91%	331%	365%
NB	$41.60B	$417.54B	$745.74B

Age- & risk-based recommendations			
Time Horizon	Year 1	Year 7	Year 15
Costs	$54.01B	$147.64B	$246.01B
VSL			
Benefits	$254.39B	$1568.01B	$2753.95B
BCR	5	11	11
ROI	371%	962%	1019%
NB	$200.38B	$1420.37B	$2507.94B
VSLY			
Benefits	$106.61B	$633.57B	$1071.53B
BCR	2	4	4
ROI	97%	329%	336%
NB	$52.60B	$485.93B	$825.52B

When recommendations for at-risk populations are included, the year 15 BCR is 4:1 (VSLY) to 11:1 (VSL). Over 15 years, a societal investment of $246 billion averts 196 025 deaths (Figure 1), yielding NBs of $2.5 trillion (VSL) or $815 billion (VSLY), respectively.

As a complementary analysis, we estimated the net present value of 1 year's vaccination activity. With approximately $22 billion spent on the annual vaccination, the programs generate $101 billion (VSLY) to $241 billion (VSL) in net benefits, with more than 18 000 deaths averted, almost 300 000 hospitalizations prevented, and more than 3 million outpatient appointments averted. This corresponds to a BCR of 6:1 (VSLY) to 12:1 (VSL). Full details of this analysis including methods, input specifications, and results are presented in Technical Appendix Section 6.3.

### Scenario analyses

**Figure 2.**
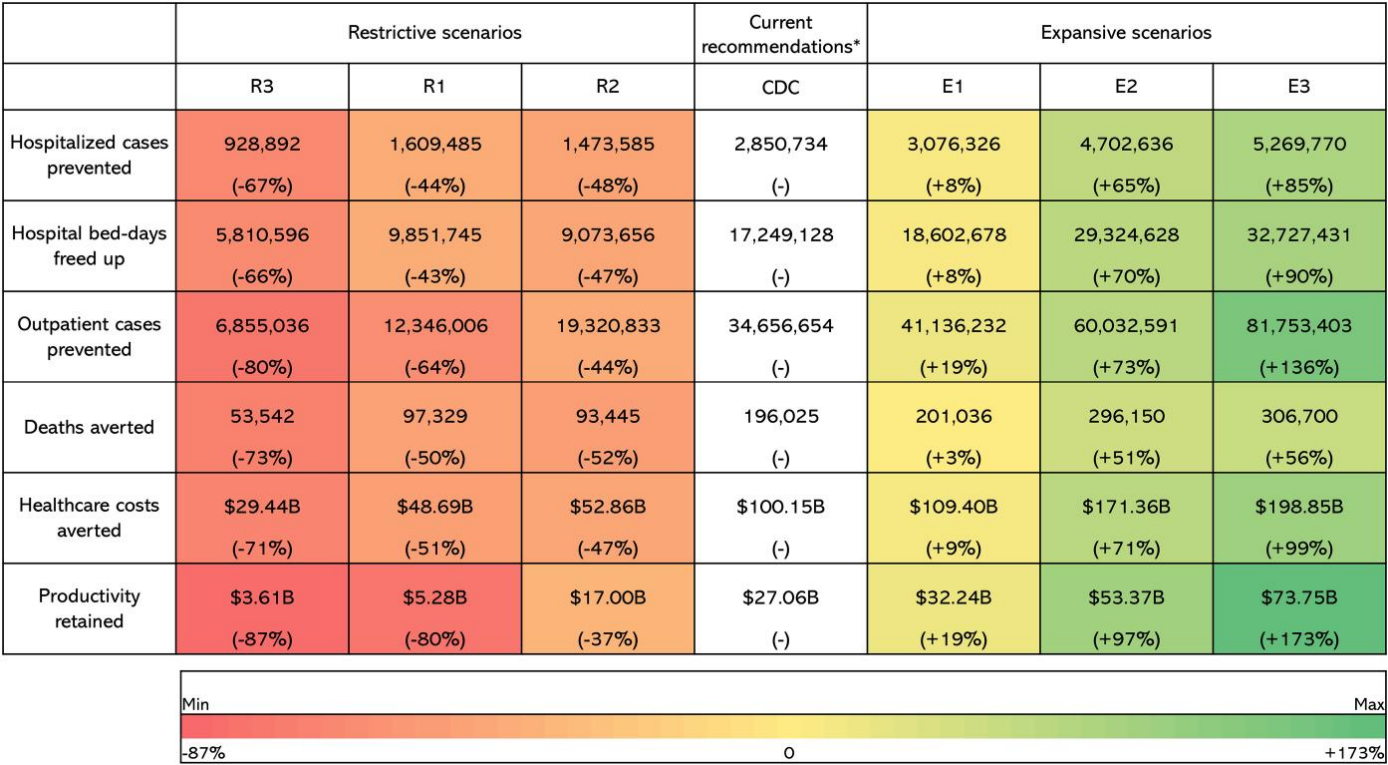
Comparison of eligibility and coverage changing policy scenarios relative to current CDC age- and risk-based recommendations at year 15.

Figure 2 shows the relative change in various medical sub-outcomes when comparing each scenario to the current CDC age- and risk-based recommendations. If age-based recommendations were restricted to those over 75 years old (scenario R1), or vaccine coverage rates exceeding 25% were to drop to 25% among all eligible adults in all four programs (scenario R2), roughly 100 000 additional vaccine-preventable deaths would occur over the 15-year horizon relative to current recommendations, with hospitalizations averted falling by 43%-48% and healthcare costs averted roughly halving. When combining these welfare-reducing scenarios of restricted eligibility and reduced coverage (scenario R3), over 140 000 additional deaths and almost 2 000 000 additional hospitalizations could occur, costing an additional $70 billion to the healthcare system.

Assuming expanded COVID-19 age-based eligibility recommendations for all adults 18+ (scenario E1), could retain $32 billion in potential productivity and prevent an additional 5000 deaths. When assuming increased coverage to internationally benchmarked targets of 75% (scenario E2), 296 150 deaths are averted, hospitalizations averted increase by 65%, and healthcare costs averted rise by 71%. Combining these assumptions (scenario E3) leads to 306 700 deaths averted by year 15, and averted productivity losses of almost $74 billion.

## Discussion

The results demonstrate that adult respiratory vaccination programs in the US generate socioeconomic benefits that are several times greater than the costs of delivering them. When mortality risk reduction is valued with age-adjusted values, the societal valuation of all benefits created by the current age-based CDC recommendations outweighs the costs by 5 times over 15 years. When mortality risk reduction is valued equally regardless of age, the societal valuation of year 15 benefits outweighs costs by a factor of 12. Overall, the results demonstrate that investments in vaccination programs yield positive returns.

The socioeconomic benefits increase if younger adults at increased risk of severe disease are vaccinated as well. Including risk-based recommendations increases net benefits by more than 6%, highlighting the value of the current CDC recommendations, and underscoring the sizeable additional societal returns associated with vaccinating adults at elevated risk of severe disease.

Maintaining the current age- and risk-based vaccination guidelines promulgated by the CDC, and sustaining these over a 15-year time period across all cohorts, will prevent almost 35 million outpatient visits and almost 3 million hospitalizations necessitating over 17 million bed days, as well as avoiding almost 200 000 vaccine-preventable deaths. This creates cost savings to the health system of more than $100 billion, while also averting almost $27 billion in productivity losses. This study reinforces the broader view that spending on preventive measures, such as vaccination, should be seen as both a public health and strategic economic investment rather than just a cost, as maintaining good health can often be achieved more cost-effectively through such preventive investments, benefitting individuals, the economy and society more broadly.^68,69^

The large returns we find are consistent with previous BCAs that show vaccination often produces benefits that substantially exceed program costs when broader societal impacts are counted, across pediatric and adult populations in the US.^20,21,58^ Our analysis expands those findings to four adult respiratory vaccination programs with the most recent data.

Given the highly positive benefit-cost profile of adult vaccination programs, which further increase with expanding eligibility and coverage, the allocated budget for vaccination may require further consideration to ensure the maximization of public health and societal welfare. The US spent roughly $4.9 trillion on health in 2023.^70^ While total vaccination spending spans multiple public and private funding streams, the CDC's Section 317 Immunization Grants Program, a key driver of coverage improvements,^71^ received just $682 million annually in recent years.^72^ Our results suggest the societal returns from adult respiratory vaccination in the first year alone far exceed this investment. Given that our model assumes high upfront year 1 costs associated with PD and RSV, this is likely a conservative estimate.

The complementary analysis of 1-year vaccination activity reframes this investment case in annual terms. Whereas our primary analysis estimates the total societal value of current CDC recommendations across all eligible cohorts over the duration of protection, the complementary analysis isolates the net present value of 1 year's vaccination activity, which is more directly comparable to the annual budgetary and programmatic cycles within which vaccine policy is made. The primary analysis quantifies what is at stake if current recommendations are sustained or weakened; the complementary analysis quantifies the annual return on continued investment. Both yield the same headline conclusion: each dollar spent on adult respiratory vaccination generates up to $12 in societal value. Realizing that value, however, depends on the policy environment in which these programs operate.

To sustain and expand these benefits, policy decisions should be grounded in rigorous scientific evidence. Clear, consistent recommendations from trusted public health institutions are essential to maintain confidence, guide efficient investment, and ensure that preventive interventions like vaccination continue to deliver broad welfare gains for society. The CDC's recommendation of co-administration of influenza, COVID-19, and RSV vaccines together is important practical guidance. Streamlining administration can both increase coverage and reduce costs, including transportation, time and productivity costs for patients and healthcare providers.^30,73^ The CDC's recent change in COVID-19 vaccination recommendations to SCDM, however, removes the clarity provided by routine recommendations. In the setting of persistent public misinformation regarding vaccine safety^51^ and declining trust in public health institutions,^47,74^ such shifts in recommendation language may unintentionally complicate communication and undermine efforts to sustain vaccine coverage.^75^ This policy development introduces uncertainty around eligibility and risks further reducing already suboptimal vaccine coverage. Evidence suggests that SCDM can lead to narrower interpretations of eligibility and lower uptake, particularly among underserved populations.^76-79^ Our scenario E1 illustrates the additional societal value that could be captured if COVID-19 vaccination extended more broadly across the adult population.

The results of our scenario analyses emphasize the risks of restricting eligibility and reducing vaccine coverage. Either narrowing age-based eligibility or allowing coverage to decline independently leads to over 100 000 additional vaccine-preventable deaths over 15 years (Figure 2). Combining both restrictions compounds these losses substantially, with significant consequences for hospitalizations and healthcare costs. Conversely, expanding eligibility and raising coverage toward international benchmarks could avert over 100 000 additional deaths beyond current levels while freeing substantial healthcare capacity.

Even modest increases in coverage produce disproportionately large societal gains. Coverage rates, particularly for RSV and COVID-19, remain low relative to comparable developed nations and WHO recommendations,^32,35^ constraining welfare gains. Raising coverage toward a 75% benchmark across the four programs could free up almost 30 million bed days and over 60 million outpatient appointments, as well as avoid more than 100 000 additional vaccine-preventable deaths relative to presently observed vaccine coverage rates. To realize these gains, policymakers should prioritize measures that directly raise and sustain coverage, including removing financial and logistical barriers to vaccination, increasing vaccine confidence, and providing outreach and support for low coverage and uninsured groups.

### Limitations

We recognize several limitations. Our primary analytic approach estimates the full societal value of current CDC recommendations by aggregating benefits across all eligible cohorts over the duration of vaccine protection. This framing has two features that warrant acknowledgement. First, the approach combines cumulative coverage rates for one-off vaccines (PD and RSV) with annual coverage rates for seasonal vaccines (influenza and COVID-19), so the coverage inputs across the four programs are not strictly comparable. Second, because benefits are aggregated across multiple programs, multiple cohorts, and a 15-year horizon, the resulting figures are large and their magnitude can be difficult to contextualize against the annual budgetary, appropriations, and programmatic cycles within which vaccine policies are typically made. The complementary analysis (Technical Appendix Section 6.3) reports the net present value of 1 year's vaccination activity in more tractable units and partially addresses both features; we recommend the two analyses be interpreted together.

Our static, closed-cohort models do not capture transmission dynamics or indirect (herd) protection, likely underestimating the total societal value of vaccination for highly transmissible respiratory pathogens. For PD specifically, omitted indirect effects, such as reduced carriage transmission and antimicrobial resistance impacts, would likely increase estimated benefits, while the potential for pediatric vaccination to reduce incremental adult benefits through serotype replacement would act in the opposite direction but is attenuated by discounting. Our models evaluate each vaccine independently and sum benefits without adjusting for overlapping eligibility, potentially double-counting averted deaths. Since mortality risk reduction largely drives benefit valuation (see sensitivity analysis in *Technical Appendix*), this may upwardly bias aggregated net benefits. We also assume that present epidemiological features, such as incidence and hospitalization rates remain constant over time. For COVID-19, we do not account for pandemic preparedness benefits or the contribution of vaccination in strengthening healthcare infrastructure for rapid response. We also exclude complications beyond the acute phase of infection, such as long-COVID or long-term sequelae of IPD, or exacerbations of underlying conditions caused by respiratory infection, thereby underestimating disease burden and the benefits of prevention. We do not include informal productivity losses or presenteeism due to limited data, so we may underestimate productivity losses. Because we assume all adults eligible for RSV and PD vaccination are vaccinated in year 1, upfront costs are inflated relative to real-world practice, which may reduce year-1 BCRs and NBs. Additionally, our analysis focuses primarily on routine adult vaccination recommendations, and does not assess maternal RSV immunization, which is also recommended by the CDC. Finally, our closed-cohort structure does not capture impacts on future population ageing or longer labor-force participation, which would further increase the value of these vaccination programs in averting productivity losses.

## Conclusions

Administering vaccinations against respiratory diseases to eligible US adults according to the current age-based recommendations generates up to $12 in societal value for every $1 invested, preventing over 182 000 deaths and yielding more than $2.3 trillion in net benefits within 15 years. Adult vaccination programs hold significant untapped potential which can be unlocked by including at-risk populations, expanding eligibility to younger individuals, and increasing coverage. As limited coverage remains a significant barrier to achieving the full health and economic benefits of vaccination in the US, policy decisions must be guided by evidence. This includes maintaining clear evidence-based recommendations, improving coverage towards international targets across the population, as well as improving access for uninsured individuals.

## Supplementary Material

qxag114_Supplementary_Data

## Appendix

**Table A1.** Figure 1 data.

Age-based recommendations			
Time Horizon	Year 1	Year 7	Year 15
Hospitalized cases prevented	228 993	1 437 703	2 524 940
Outpatient cases prevented	2 772 335	16 883 909	30 722 185
Deaths averted	16 074	101 784	182 419
Healthcare costs averted	$8.07B	$49.95B	$86.90B
**Age- & risk-based recommendations**			
Hospitalized cases prevented	267 060	1 631 511	2 850 734
Outpatient cases prevented	3 075 561	18 528 404	34 656 654
Deaths averted	18 050	111 522	196 025
Healthcare costs averted	$10.00B	$58.68B	$100.15B
