# Supplementary material for: Socioeconomic value of adult respiratory vaccination in the United States: a benefit-cost analysis
Source: Health Aff Sch. 2026 May 12;4(7):qxag114. doi: 10.1093/haschl/qxag114 (PMC13384430; doi:10.1093/haschl/qxag114)
Supplement: qxag114_Supplementary_Data [file qxag114_supplementary_data.zip › US Technical Appendix_CLEAN.docx]

Technical Appendix

Contents

[1. Intro/Objectives 2](#_Toc228348074)

[2. General modelling approach and structure 2](#_Toc228348075)

[3. BCA Framework 2](#_Toc228348076)

[3.1 Costs 3](#_Toc228348077)

[3.2 Benefits 3](#_Toc228348078)

[4. Detailed Modelling Approach 5](#_Toc228348079)

[5. Disease-Specific Model Overview 5](#_Toc228348080)

[5.1 Pneumococcal Disease (PD) 5](#_Toc228348081)

[5.2 Respiratory Syncytial Virus (RSV) 9](#_Toc228348082)

[5.3 Influenza (Flu) 12](#_Toc228348083)

[5.4 COVID-19 15](#_Toc228348084)

[5.5 Comparative contextualization of epidemiological inputs 17](#_Toc228348085)

[6. Detailed results 19](#_Toc228348086)

[6.1. Full program value (age-based recommendations) 19](#_Toc228348087)

[6.2. Full program value (age- and risk-based recommendations) 20](#_Toc228348088)

[6.3. Complementary analysis: net present value (NPV) associated with one year of vaccination activity 21](#_Toc228348089)

[7. Sensitivity analyses & robustness checks 22](#_Toc228348090)

[7.1. One-way sensitivity analyses results 22](#_Toc228348091)

[7.2. Human capital valuation of mortality 24](#_Toc228348092)

[7.3. Human capital valuation of productivity 26](#_Toc228348093)

[8. References 28](#_Toc228348094)

## Intro/Objectives

This Technical Appendix outlines the methods used with the Benefit-Cost Analysis (BCA) from a societal perspective to estimate the aggregate costs and benefits of four adult respiratory vaccination programs in the US, compared to a no-vaccination or no-booster scenario. The programs analyzed include Pneumococcal Disease (PD), Respiratory Syncytial Virus (RSV), Influenza (Flu), and COVID-19.

A detailed list of data inputs and sources is provided in Tables 1-4. Results of the one-way sensitivity analysis are provided in section 5.

## General modelling approach and structure

Our general approach utilizes a **life-table-based modelling framework**, similar to the Papillomavirus Rapid Interface for Modelling and Economics (PRIME) developed by Jit et al. (2014), which is particularly useful for evaluating vaccination programs on a population level.

The economic evaluation of choice is a **Benefit-Cost Analysis (BCA)** and is based on the Reference Case Guidelines recommended by Robinson et al. (2019). We calculate the net benefit (NBs) and benefit-cost ratio (BCR) as the key outputs. Results are presented across four key timeframes: Year 1, Year 7, Year 15 and lifetime.

We developed **four static, deterministic disease models** that follow the model structures outlined in Talbird et al. (2021) and are directly modelled on the national lifetable. A static model assumes that the intervention's impact is limited to the individuals directly affected and does not account for dynamic factors such as disease transmission patterns, herd immunity, or changing population demographics. As such, it is a conservative approach which does not fully capture the effects of herd immunity. In a deterministic model, outcomes are calculated using fixed input values without incorporating variability or uncertainty in model parameters. This produces a single set of outcomes based on point estimates.

The implementation of the four disease models is tailored to the specifics of the individual vaccination program.

The four disease models are implemented as multi cohort models and are tailored to the specifics of the individual vaccination program. Flu and COVID-19 are annual vaccination programs, so each age cohort at and above the vaccination age is vaccinated every year and followed until the age of 100 or death. RSV and PD are single-dose programs, where everyone eligible receives one dose of vaccination at year 1 and no revaccination occurs. RSV offers protection for 7 years, and PD offers protection for 15 years.

In summary, our approach allows us to track the vaccination cohorts associated with each program in the US over time, estimate all-cause and disease-specific mortality, and identify relevant health outcomes at different stages of life to quantify the monetized costs and benefits from a societal perspective.

## BCA Framework

We followed and adapted an established benefit-cost analysis (BCA) framework to evaluate the four programs from a societal perspective, using the Reference Case Guidelines recommended by Robison et al. (2019) to structure the valuation of costs and benefits.

This framework distinguishes between monetary inputs (e.g., costs of an immunization program) and monetized outputs (e.g., gains in health and wealth) to evaluate a policy option's benefit-cost profile. The guidelines also outline various approaches to monetizing health benefits, which differ in their consideration of mortality and morbidity and in their data requirements.

The main outputs of the BCA are the benefit-cost ratio (BCR) and net benefits (NBs). BCR is the ratio between the total benefits, the monetary value of all benefits generated by the program, and the total costs, the full cost of the program. A BCR >1 indicates that the program generates more benefits than costs. A BCR = 1 indicates that the program exactly breaks even. A BCR < 1 indicates that the program is more costly than the benefits generated.

$$BCR=\frac{Total benefits}{Total costs}$$

The NB is calculated by subtracting the total costs from the total benefits and a NB greater than 0 indicates net value generation.

Figure 1 benefit-cost analysis framework

Adapted based on Robinson et al. (2019)


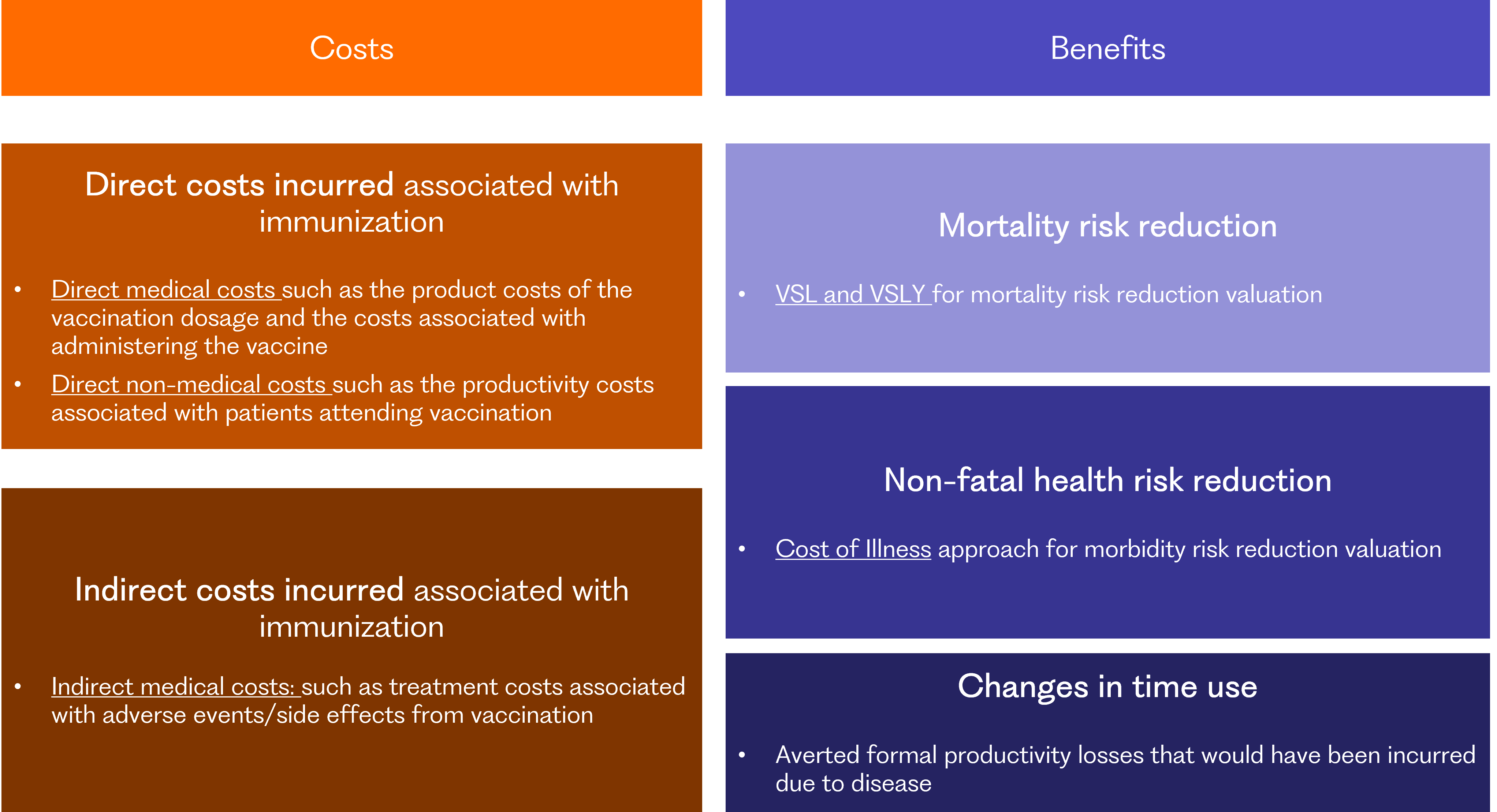


### 3.1 Costs

The analysis includes direct medical and non-medical costs associated with delivering the immunization program along with indirect costs. It excludes the initial setup costs of the program as the US’ established immunization infrastructure minimizes additional implementation expenses. Given that foundational investments are largely sunk costs and ongoing delivery costs are more relevant for assessing cost-effectiveness, this approach aligns with standard health economic evaluation practices.

Direct medical costs are mainly comprised of vaccine dosage costs and are included in all programs. Direct non-medical costs include vaccine administration and productivity losses due to vaccination. For the latter, these losses are valued using hourly wages adjusted for labor force participation and unemployment rates, assuming two hours for all vaccinations in which work productivity is lost. These likely overestimate productivity losses, as some individuals may get vaccinated outside working hours or during other appointments. However, given the uncertainty, we adopted a simplified approach, assuming all employed adults use working hours for vaccination.

We adopt a conservative approach by incorporating vaccine-attributable adverse event costs as additional costs rather than offsets to benefits. Long-term or serious adverse events are excluded due to their low probability. To mitigate this, we assume a minor adverse event for all vaccinations that requires a standard adult painkiller dosage proxied by the value of two common painkiller (paracetamol) tablets per vaccine dose.

While these costs are minimal and have a marginal impact, this method lowers the benefit-cost ratio (BCR) by capturing the effect on the denominator rather than the numerator.

All costs are inflated/deflated to and presented in USD 2024. Benefits are discounted at 2% p.a. as advised by The White House, (2023).

### 3.2 Benefits

All benefits are captured within three outcomes ‘buckets’:

1. Reductions in mortality risk
2. Reductions in morbidity risk
3. Changes in productivity loss of the affected individuals.

#### 3.2.1. Mortality

To monetize reductions in mortality risk, we use two methods.

Method 1 assumes that all mortality risk reductions should be valued equally, regardless of an individual's age. Method 2 assumes that the value of reducing mortality risk decreases as individuals approach the end of their natural lifespan.

For method 1, we value each fatal case with the Value of a Statistical Life (VSL), which provides a single value for preventing the loss of a life, irrespective of the remaining life expectancy and hence treats all equally. For method 2, we use the Value of a Statistical Life Year (VSLY), which can be derived from the VSL. This assigns a value to each additional year of life gained, which leads to an adjustment of the VSL depending on when a person dies. This is the recommended adjustment made to the VSL for programs that may affect younger or older populations predominantly (Robinson et al., 2019). Together, the VSL and VSLY valuations provide a reasonable upper (VSL) and lower (VSLY) estimate of the value that society places on mortality risk reduction, ensuring results are robust via different valuation methods.

We use a US VSL of $13.4 million (Kearsley, 2025) from which we also derive the VSLY. The steps used to estimate this are described below.

Firstly, based on US life table data, we estimate the person-years lived between ages $x$ and $x+1$ ($L_{x}(i)$) for each age ($i$) to 100. This is calculated by taking the average of the proportion of the total cohort surviving to age ($l_{x}(i)$) $x$ and $x+1$:

$$L_{x}\left( i \right)=\frac{(l_{x}+l_{x+1})}{2}$$

Next, we derive the total number of person-years lived above age $x$ ($T_{x}(i)$). For each age ($i$) to 100, we take the summation of $L_{x}\left( i \right)$ for all ages above $x$.

Following this, we estimate the expected years of life remaining at age $x$($E_{x}(i)$), for each age ($i$) up to 100. This is estimated by:

$$E_{x}\left( i \right)=\frac{T_{x}}{l_{x}}$$

From this, the age at which an individual has half of their expected life years remaining is estimated. In the US, the median age is 41 years. The VSL of $13.4 million is then divided by the undiscounted remaining life expectancy at this median age (nearly 40 years) to derive the VSLY of $0.35 million.

#### 3.2.2. Morbidity

Morbidity risk reduction is monetized using a cost-of-illness approach capturing the relevant age- and risk-group-specific healthcare resources and utilization, including hospital stays and outpatient visits (including GP appointments and prescriptions).

#### 3.2.3. Changes in time use

Productivity loss changes are estimated by calculating the avoided formal productivity losses from averted illness, based on the average number of workdays lost per outcome multiplied by the average daily wage. Labor force participation rates were collected from the U.S. Bureau of Labor Statistics (U.S. Bureau of Labor Statistics, 2025a; b).

We calculated an adjusted population employment rate by multiplying the labor force participation rate by the reciprocal of the unemployment rate. Formal productivity losses from averted premature mortality are excluded as they are captured in the VSL value. Informal productivity losses are also excluded due to limited data, potentially underestimating productivity losses, particularly in older age groups beyond retirement age.

## 4. Detailed Modelling Approach

We model the vaccination course across age-based cohorts (and risk-based cohorts where recommended) using four disease-specific, closed multi-cohort models that are based on national lifetables, which is similar to the approach taken by others (Jit et al., 2014).

The model distinguishes two arms: vaccinated and unvaccinated. Each arm follows the respective age-cohort age cohorts over their lifetime or the maximum age of 100, incorporating life table data to estimate the probability of death between consecutive ages (X and X+1).

In the vaccinated arm, vaccine coverage and vaccine effectiveness (or efficacy) are applied. The difference between the two arms provides the number of events prevented by vaccination compared to a no-vaccination scenario.

Within each disease model, we estimate all relevant disease-specific outcomes by individual age cohorts using different parameter inputs reflecting different risks at different ages or between the general and the at-risk populations. For PD, these outcomes include Meningitis hospitalizations, Bacteremia hospitalizations, NBPP hospitalizations, and NBPP outpatient (no cases are assumed to be not medically attended (NMA). For RSV, flu and COVID-19, these include hospitalization, outpatient cases, as well as NMA cases for Flu and COVID, and emergency department cases for Flu only.

For all cohorts, the number of events is aggregated over the remaining lifetime:

$$Num\_Events =\frac{{Incidence\_Event\_Adj}_{Age,Risk}\times{Incidence\_Event}_{Age, Risk}}{100,000}$$

We then calculate the number of deaths per event, based on the number of events and the proportion of cases that lead to death:

$${Event\_Deaths= Num\_Events}_{Age, Risk}\times{Event\_Mortality\_Rate}_{Age, risk}$$

Next, we calculate the expected life years lost per person event and the number of deaths.

$${LYL\_Outcome= Event\_Deaths}_{Age, Risk}\times{Exp\_Remaining\_LYs}_{Age}$$

All outcomes are then monetized. Outpatient and inpatient appointments are valued with age and outcome-specific (and risk-specific where relevant) medical costs, while productivity losses (see sections 3.2.2 and 3.3.3) are computed based on workdays lost associated with each of the four outcomes and age-specific wage rates.

The number of deaths and life years lost are monetized using the VSL or VSLY.

$$Monetised\_Mortality\_VSL=VSL\times Event\_Deaths$$

$$Monetised\_Mortality\_VSLY=VSLY\times LYL\_Outcome$$

## 5. Disease-Specific Model Overview

### 5.1 Pneumococcal Disease (PD)

#### 5.1.1 Model description

The pneumococcal disease (PD) model is implemented as a multi-cohort model, tracking age- and risk-based cohorts from the time of vaccination until age 100 or death. The model runs in annual cycles with the age-based cohort beginning at age 50 and running for up to 50 years (until age 100), while the risk-based cohort starts at age 18 and runs for up to 82 years. All individuals in the modelled cohorts are vaccinated at year 1 and no revaccination occurs. Results from both cohorts are combined to estimate the total population effects within the PD model. To calculate aggregate results across all four programs, results from each disease model are weighted by the relative population size.

The model for PD follows the general decision tree used across the four programs, adapted from Talbird et al (2021) with some disease-specific differences.

Figure 2 PD decision tree model


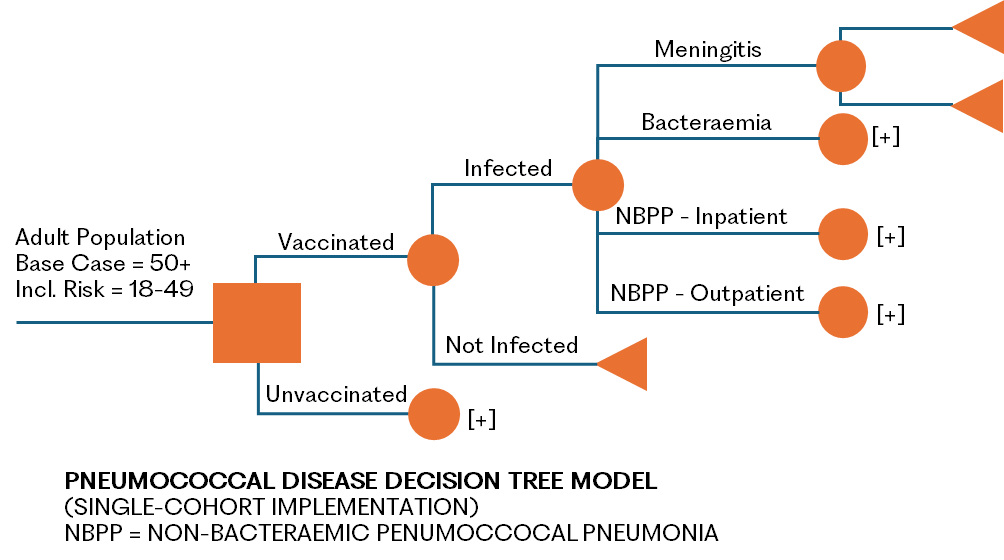


#### 5.1.2 Vaccination program specifications

We model a 20-valent pneumococcal conjugate vaccine (PCV20), which targets 20 of the most clinically relevant serotypes responsible for pneumococcal disease.

The PD model uses disease-specific outcomes, in contrast to the more general outcomes used in the other three programs. These outcomes are due to the potential for infectious diseases caused by the Streptococcus pneumoniae bacterium to manifest as the more severe forms of invasive pneumococcal disease (IPD), including meningitis and bacteremia, or as non-bacteremic pneumococcal pneumonia (NBPP) in both the inpatient and outpatient setting, as detailed below.

- (IPD) Meningitis
- (IPD) Bacteremia
- (NBPP) – Inpatient
- (NBPP) – Outpatient

These outcomes are reflected as parameters in the full input table found in Table 1.

We collected age- and risk-specific incidence rates and case fatality rates in the vaccine naive cohort and adjusted the incidences by vaccine-type specific serotype by multiplying with an adjustment factor. This adjustment represents the serotype distribution of IPD in the US for the vaccine being modelled. We assumed the same adjustment factor applied for NBPP (Table 1).

We model one complete vaccination course based on one dose in the base year and assume specific vaccine efficacy rates against the invasive pneumococcal disease (IPD) and non-bacteremic pneumococcal pneumonia (NBPP) outcomes. We aimed to find age- and risk-specific coverage rates where possible and used the most current recommendation for the US’ National Immunization Program (NIP).

Initial vaccine efficacy was assumed to persist for the first 5 years with no observed waning. Following this vaccine waning rates were calculated by an annual linear decline from years 6-15. From year 16 onwards the vaccine was assumed to no longer provide protection against PD.

Table 1 PD parameters

*Costs are reported in USD2024.*

| **Parameter** | **Value** | **Source** |
| --- | --- | --- |
| Vaccination age (age-based) | 50 | (CDC, 2025g) |
| Total population cohort size (age based) | 123,737,699 | (United Nations, Department of Economic and Social Affairs, Population Division, 2024) |
| Vaccine Coverage % (age-based) | 64.00% | (CDC, 2025j) |
| Vaccine initial reduction in IPD cases % | 75.00% | (Bonten et al., 2015; CDC, 2025g) |
| Vaccine IPD reduction efficacy decrease annually % | 7.50% | (Gourzoulidis et al., 2023) |
| Vaccine initial reduction in NBPP cases % | 45.00% | (Bonten et al., 2015; CDC, 2025g) |
| Vaccine NBPP reduction efficacy decrease annually % | 4.50% | (Gourzoulidis et al., 2023) |
| IPD incidence adjustment for vaccine serotypes 50-64 | 58% | (CDC, 2024a) |
| IPD incidence adjustment for vaccine serotypes 65+ | 54% |  |
| NBPP incidence adjustment for vaccine serotypes 50-64 | 58% |  |
| NBPP incidence adjustment for vaccine serotypes 65+ | 54% |  |
| Meningitis Incidence for unvaccinated 50-64 per 100,000 | 1.10 | (Talbird et al., 2021) |
| Meningitis Incidence for unvaccinated over 65 per 100,000 | 2.20 |  |
| Bacteremia Incidence for unvaccinated 50-64 per 100,000 | 16.30 |  |
| Bacteremia Incidence for unvaccinated over 65 per 100,000 | 32.30 |  |
| Pneumonia (NBPP) Incidence for unvaccinated 50-64 per 100,000 | 257.50 |  |
| Pneumonia (NBPP) Incidence for unvaccinated over 65 per 100,000 | 1,015.60 |  |
| Pneumonia Inpatient 50-64 case % | 30.10% |  |
| Pneumonia Inpatient 65+ case % | 40.60% |  |
| Pneumonia Outpatient 50-64 case % | 69.90% |  |
| Pneumonia Outpatient 65+ case % | 59.40% |  |
| Meningitis/bacteremia cases resulting in death 50-64 % | 10.70% |  |
| Meningitis/bacteremia cases resulting in death 65+ % | 14.90% |  |
| NBPP inpatient cases resulting in death 50-64 % | 3.20% |  |
| NBPP inpatient cases resulting in death 65+ % | 6.70% |  |
| NBPP outpatient cases resulting in death 50-64 % | 0.00% |  |
| NBPP outpatient cases resulting in death 65+ % | 0.00% |  |
| Vaccine Acquisition Cost per Recommended Dosage (age-based) | $274.60 | (CDC, 2025c) |
| Vaccine Administration Cost per Recommended Dosage (age-based) | $31.59 | (Leidner and Bletnitsky, 2024) |
| Vaccine-related Adverse Event Cost per Recommended Dosage (age-based) | $0.20 | (Rite Aid, 2024) |
| Cost of Productivity Loss due to attending vaccination (age-based) | $52.77 | See section 3.1 |
| Meningitis/bacteremia direct medical costs per case 50-64 | $56,057.37 | (Talbird et al., 2021) |
| Meningitis/bacteremia direct medical costs per case 65+ | $37,822.11 |  |
| Meningitis/bacteremia indirect costs per case 50-64 | $3,050.09 | See section 3.2.3 |
| Meningitis/bacteremia indirect costs per case 65-74 | $894.21 |  |
| Meningitis/bacteremia indirect costs per case 75-84 | $0.00 |  |
| Meningitis/bacteremia indirect costs per case 85+ | $0.00 |  |
| NBPP Inpatient direct medical costs per case 50-64 | $48,780.4 | (Talbird et al., 2021) |
| NBPP Inpatient direct medical costs per case 65+ | $32,516.19 |  |
| NBPP Inpatient indirect costs per case 50-64 | $2,174.11 | See section 3.2.3 |
| NBPP Inpatient indirect costs per case 65-74 | $637.39 |  |
| NBPP Inpatient indirect costs per case 75-84 | $0.00 |  |
| NBPP Inpatient indirect costs per case 85+ | $0.00 |  |
| NBPP outpatient direct medical costs per case 50-64 | $177.52 | (Talbird et al., 2021) |
| NBPP outpatient direct medical costs per case 65+ | $355.03 |  |
| NBPP outpatient indirect costs per case 50-64 | $1,097.61 | See section 3.2.3 |
| NBPP outpatient indirect costs per case 65-74 | $321.79 |  |
| NBPP outpatient indirect costs per case 75-84 | $0.00 |  |
| NBPP outpatient indirect costs per case 85+ | $0.00 |  |
| Vaccination age (at-risk) | 18 | (CDC, 2025g) |
| Total population cohort size (at-risk) | 34,804,962 | (United Nations, Department of Economic and Social Affairs, Population Division, 2024) |
| Vaccine Coverage % (at-risk) | 23.00% | (Wateska et al., 2020) |
| IPD incidence adjustment for vaccine serotypes 18-49 per 100,000 (at-risk) | 62.1% | (King et al., 2024) |
| NBPP incidence adjustment for vaccine serotypes 18-49 per 100,000 (at-risk) | 62.1% | (Wateska et al., 2020) |
| Meningitis Incidence for unvaccinated 18-49 per 100,000 (at-risk) | 0.45 | (Grant et al., 2023; Marrie et al., 2017; Oligbu et al., 2019) |
| Bacteremia Incidence for unvaccinated 18-49 per 100,000 (at-risk) | 8.80 |  |
| Pneumonia (NBPP) Incidence for unvaccinated 18-49 per 100,000 (at-risk) | 316.80 | (Wateska et al., 2020) |
| Pneumonia Inpatient case 18-49 (at-risk) % | 63% |  |
| Pneumonia Outpatient case 18-49 (at-risk) % | 37% |  |
| Meningitis/bacteremia cases resulting in death 18- 49 (at-risk) % | 6.22% | (Owusu-Edusei, Deb and Johnson, 2022; Mendes et al., 2022) |
| NBPP inpatient cases resulting in death 18- 49 (at-risk) % | 6.4% |  |
| NBPP outpatient cases resulting in death 18- 49 (at-risk) % | 0.00% | Assumption |
| Vaccine Acquisition Cost per Recommended Dosage (at-risk) | $274.60 | (CDC, 2025c) |
| Vaccine Administration Cost per Recommended Dosage (at-risk) | $31.59 | (Leidner and Bletnitsky, 2024) |
| Vaccine-related Adverse Event Cost per Recommended Dosage (at-risk) | $0.20 | (Rite Aid, 2024) |
| Cost of Productivity Loss due to attending vaccination (at-risk) | $56.36 | See section 3.1 |
| Meningitis/bacteremia direct medical costs per case 18- 49 (at-risk) | $56,057.37 | (Talbird et al., 2021) |
| Meningitis/bacteremia indirect costs per case 18-49 (at-risk) | $3,257.58 | See section 3.2.3 |
| NBPP Inpatient direct medical costs per case 18- 49 (at-risk) | $48,780.4 | (Talbird et al., 2021) |
| NBPP Inpatient indirect costs per case 18-49 (at-risk) | $2,322.01 | See section 3.2.3 |
| NBPP outpatient direct medical costs per case 18- 49 (at-risk) | $177.52 | (Talbird et al., 2021) |
| NBPP outpatient indirect costs per case 18-49 (at-risk) | $1,172.28 | See section 3.2.3 |

### 5.2 Respiratory Syncytial Virus (RSV)

#### 5.2.1 Model description

The Respiratory Syncytial Virus (RSV) model is implemented as a multi-cohort model, tracking annual cohorts from the time of vaccination until age 100 or death.

It operates on annual cycles. We model one vaccination course for all individuals over the age of 75, as well as one course for risk-based groups between the ages of 50 and 74 years. As in other programs, at-risk individuals included those with comorbid conditions (moderate risk) as well as immunocompromised (high risk).

The model follows the general decision tree used across the four programs adapted from Talbird et al. (2021), as shown in Figure 3. While the decision-tree includes ED, we do not generally use this branch as we capture ED (and ICU) incidences, outcomes and costs by adjusting to include them within hospitalization.

Figure 3 RSV decision tree model


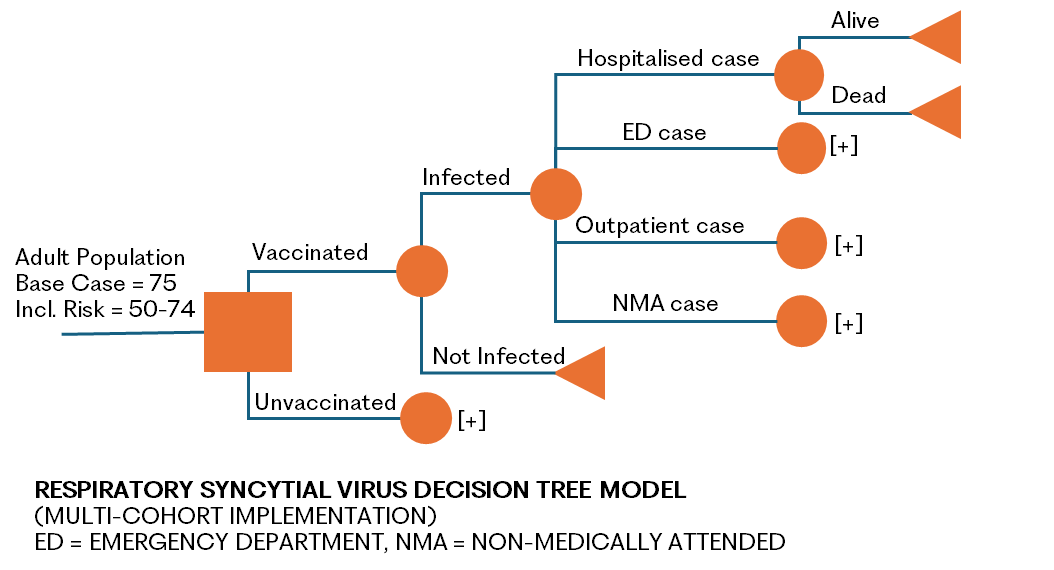


#### 5.2.2. Vaccination program specifications

As with the other programs, we aimed for age- and risk-specific incidence and case fatality rates in the absence of vaccination. We applied yearly average specific vaccine efficacies for the modelled outcomes, incorporating separate waning profiles against each outcome. VE against hospitalization wanes annually from year 1 to reach 0 by year 7 (7 years of protection), while VE against outpatient and NMA wanes annually from year 1 to reach 0 by year 5 (5 years of protection).

Full details and sources for all RSV parameters are detailed in Table 2, below. Costs are reported in USD2024.

Table 2 RSV parameters

*Costs are reported in USD2024.*

| **Parameter** | **Value** | **Source** |
| --- | --- | --- |
| Vaccination Age (age-based) | 75 | (CDC, 2025h) |
| Total population cohort size (age-based) | 24,984,060 | (United Nations, Department of Economic and Social Affairs, Population Division, 2024) |
| Cohort Vaccine Coverage % (age-based) | 21.33% | (La et al., 2025) |
| Vaccine Efficacy Hospitalization/ED Year 1 Weighted Average | 82.54% | Calculated using vaccine effectiveness data from Tartof et al., (2025b; a), applying linear waning beyond 13 months |
| Vaccine Efficacy Hospitalization/ED Year 2 Weighted Average | 70.79% |  |
| Vaccine Efficacy Hospitalization/ED Year 3 Weighted Average | 59.05% |  |
| Vaccine Efficacy Hospitalization/ED Year 4 Weighted Average | 47.30% |  |
| Vaccine Efficacy Hospitalization/ED Year 5 Weighted Average | 35.56% |  |
| Vaccine Efficacy Hospitalization/ED Year 6 Weighted Average | 23.81% |  |
| Vaccine Efficacy Hospitalization/ED Year 7 Weighted Average | 12.07% |  |
| Vaccine Efficacy Outpatient Year 1 Weighted Average | 62.69% | Calculated using vaccine efficacy data from Walsh et al., (2023), applying the effect of waning from (Averin et al., 2025a) |
| Vaccine Efficacy Outpatient Year 2 Weighted Average | 42.96% |  |
| Vaccine Efficacy Outpatient Year 3 Weighted Average | 19.85% |  |
| Vaccine Efficacy Outpatient Year 4 Weighted Average | 1.48% |  |
| Vaccine Efficacy NMA Year 1 Weighted Average | 56.20% |  |
| Vaccine Efficacy NMA Year 2 Weighted Average | 25.01% |  |
| Vaccine Efficacy NMA Year 3 Weighted Average | 0.62% |  |
| Vaccine Efficacy NMA Year 4 Weighted Average | 0.00% |  |
| Hospitalized case incidence per 100,000 older adults | 462 | (Averin et al., 2024) |
| ED case incidence per 100,000 older adults | 307 |  |
| Outpatient case incidence per 100,000 older adults | 3,240 |  |
| NMA case incidence per 100,000 older adults | 5,932 | Calculated as 67.6% of total RSV cases reported as NMA from Herring et al. (2022) applied to case numbers from Averin et al. (2024) |
| Deaths from hospitalized cases % older adults | 9.78% | (Averin et al., 2024) |
| Deaths from ED cases % older adults | 0.00% | Assumption |
| Deaths from outpatient cases % older adults | 0.00% |  |
| Deaths from NMA cases % older adults | 0.00% |  |
| Vaccine Acquisition Cost per Recommended Dosage (age-based) | $306.80 | (CDC, 2025c) |
| Vaccine Administration Cost per Recommended Dosage (age-based) | $25.60 | (Moghadas et al., 2024) |
| Vaccine-related Adverse Event Cost per Recommended Dosage (age-based) | $0.20 | (Rite Aid, 2024) |
| Cost of Productivity Loss due to attending vaccination (age-based) | $15.48 | See section 3.1 |
| Direct medical cost per case hospitalized case (age-based) | $22,753.72 | (Averin et al., 2024) |
| Direct medical cost per case ED case (age-based) | $3,158.55 |  |
| Direct medical cost per case outpatient case (age-based) | $980.33 |  |
| Direct medical cost per case NMA case (age-based) | $0.00 | Assumption |
| Indirect (productivity) cost per hospitalized case 75+ | $1,045.20 | See section 3.2.3 |
| Indirect (productivity) cost per ED case 75+ | $215.19 |  |
| Indirect (productivity) cost per outpatient case 75+ | $153.71 |  |
| Indirect (productivity) cost per NMA case 75+ | $61.48 |  |
| Vaccination Age (at-risk) | 50 | (CDC, 2025h) |
| Total population cohort size (at-risk) | 58,359,537 | (United Nations, Department of Economic and Social Affairs, Population Division, 2024) |
| Cohort Vaccine Coverage % (at-risk) | 15.29% | (La et al., 2025) |
| Hospitalized case incidence per 100,000 (at-risk) | 418 | (Averin et al., 2024) |
| ED case incidence per 100,000 (at-risk) | 271 | (Averin et al., 2024) |
| Outpatient case incidence per 100,000 (at-risk) | 3,288 |  |
| NMA case incidence per 100,000 (at-risk) | 5,884 | Calculated as 67.6% of total RSV cases reported as NMA from Herring et al. (2022) applied to case numbers from Averin et al. (2024) |
| Deaths from hospitalized cases (at-risk) % | 5.34% | (Averin et al., 2024) |
| Deaths from ED cases (at-risk) % | 0.00% | Assumption |
| Deaths from outpatient cases (at-risk) % | 0.00% |  |
| Deaths from NMA cases (at-risk) % | 0.00% |  |
| Vaccine Acquisition Cost per Recommended Dosage (at-risk) | $306.80 | (CDC, 2025c) |
| Vaccine Administration Cost per Recommended Dosage (at-risk) | $25.60 | (Moghadas et al., 2024) |
| Vaccine-related Adverse Event Cost per Recommended Dosage (at-risk) | $0.20 | (Rite Aid, 2024) |
| Cost of Productivity Loss due to attending vaccination (at-risk) | $52.77 | See section 3.1 |
| Direct medical cost per case hospitalized case (at-risk) | $39,647.60 | (Averin et al., 2024) |
| Direct medical cost per case ED case (at-risk) | $6,564.38 |  |
| Direct medical cost per case outpatient case (at-risk) | $1,096.35 |  |
| Indirect (productivity) cost per hospitalized case 50-64 | $3,588.34 | See section 3.2.3 |
| Indirect (productivity) cost per hospitalized case 65+ | $1,052.01 |  |
| Indirect (productivity) cost per ED case 50-64 | $738.78 |  |
| Indirect (productivity) cost per ED case 65+ | $216.59 |  |
| Indirect (productivity) cost per outpatient case 50-64 | $527.70 |  |
| Indirect (productivity) cost per outpatient case 65+ | $154.71 |  |
| Indirect (productivity) cost per NMA case 50-64 | $105.54 |  |
| Indirect (productivity) cost per NMA case 65+ | $61.88 |  |

5.3 Influenza (Flu)

5.3.1 Model description

The Influenza (Flu) model is implemented as a multi-cohort model, tracking annual cohorts from the time of vaccination until age 100 or death. In the base case, the cohort begins at age 18 and runs in annual cycles for 82 years. We do not model a separate risk group, everyone over the age of 18 is eligible for a vaccination each year.

The model follows the general decision tree used across the four programs adapted from Talbird et al. (2021), as shown in Figure 4. While the decision-tree includes ED, we do not generally use this branch as we capture ED (and ICU) incidences, outcomes and costs by adjusting to include them within hospitalization.

Figure 4 Flu decision tree model


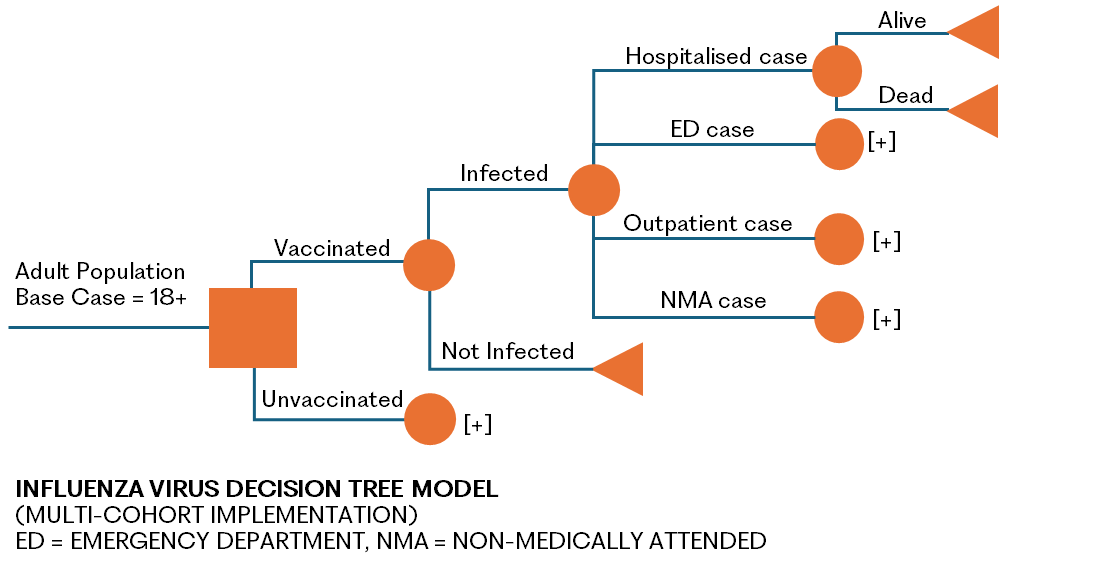


#### 5.3.2 Vaccination program specifications

Vaccine efficacy against influenza cases is calculated based on averages from CDC data (CDC, 2024c)

Due to the seasonality of flu cases, we assume that most flu cases and thus protection against them occur within narrow periods each year, with every subsequent vaccination protecting against the next flu season.

To collect representative incidence rates, we excluded values found in the years 2021 and 2022, to exclude the effect of COVID-19.

Full details and sources for all Flu parameters are detailed in Table 3, below.

Table 3 Flu parameters

*Costs are reported in USD2024.*

| **Parameter** | **Value** | **Source** |
| --- | --- | --- |
| Vaccination Age | 18 | (Talbird et al., 2021) |
| Total population cohort size | 269,365,153 | (United Nations, Department of Economic and Social Affairs, Population Division, 2024) |
| Cohort 18-49 Vaccine Coverage % | 32.72% | (CDC, 2024b) |
| Cohort 50-59 Vaccine Coverage % | 45.68% |  |
| Cohort 60-64 Vaccine Coverage % | 45.68% |  |
| Cohort 65+ Vaccine Coverage % | 65.87% |  |
| Vaccine Efficacy (% reduction in influenza cases) all cohorts | 35.47% | (CDC, 2025e) |
| Incidence per 100,000 unvaccinated 18–49-year-olds | 8,400 | (US Census Bureau, 2025; CDC, 2025f; d, 2024b) |
| Incidence per 100,000 unvaccinated 50–59-year-olds | 14,090 |  |
| Incidence per 100,000 unvaccinated 60–64-year-olds | 1,4090 |  |
| Incidence per 100,000 unvaccinated 65+ year olds | 7,500 |  |
| Hospitalized cases 18–49-year-olds % | 0.77% |  |
| Hospitalized cases 50–59-year-olds % | 0.77% |  |
| Hospitalized cases 60–64-year-olds % | 0.77% |  |
| Hospitalized cases 65+ year olds % | 4.90% |  |
| Emergency department cases 18–49-year-olds % | 2.60% | (Talbird, Poston and Hogea, 2017) |
| Emergency department cases 50-9-year-olds % | 2.60% |  |
| Emergency department cases 60–64-year-olds % | 2.60% | Talbird, Poston and Hogea, 2017) |
| Emergency department cases 65+ year olds % | 0.50% |  |
| Outpatient cases 18–49-year-olds % | 39.48% | (CDC, 2025d) |
| Outpatient cases 50–59-year-olds % | 39.48% |  |
| Outpatient cases 60–64-year-olds % | 39.48% |  |
| Outpatient cases 65+ year olds % | 56.00% |  |
| Nonmedically attended cases 18–49-year-olds % | 57.15% | Calculated as the remainder of 100% - hospitalization, ED and outpatient cases |
| Nonmedically attended cases 50–59-year-olds % | 57.15% |  |
| Nonmedically attended cases 60–64-year-olds % | 57.15% |  |
| Nonmedically attended cases 65+ year olds % | 38.60% |  |
| Deaths from hospitalized cases for 18–49-year-olds % | 4.08% | (Reed et al., 2015) |
| Deaths from hospitalized cases for 50–59-year-olds % | 4.08% |  |
| Deaths from hospitalized cases for 60–64-year-olds % | 4.08% |  |
| Deaths from hospitalized cases for 65+ year olds % | 7.70% |  |
| Deaths from ED cases for 18–49-year-olds % | 0.01% | (Talbird et al., 2021) |
| Deaths from ED cases for 50–59-year-olds % | 0.01% |  |
| Deaths from ED cases for 60–64-year-olds % | 0.01% |  |
| Deaths from ED cases for 65+ year olds % | 0.10% |  |
| Deaths from outpatient cases for 18–49-year-olds % | 0.01% | (Reed et al., 2015) |
| Deaths from outpatient cases for 50–59-year-olds % | 0.01% |  |
| Deaths from outpatient cases for 60–64-year-olds % | 0.01% |  |
| Deaths from outpatient cases for 65+ year olds % | 0.36% |  |
| Deaths from NMA cases for 18–49-year-olds % | 0.01% |  |
| Deaths from NMA cases for 50–59-year-olds % | 0.01% |  |
| Deaths from NMA cases for 60–64-year-olds % | 0.01% |  |
| Deaths from NMA cases for 65+ year olds % | 0.10% |  |
| Vaccine Acquisition Cost per Recommended Dosage | $15.87 | (CDC, 2025c) |
| Vaccine Administration Cost per Recommended Dosage | $20.64 | (CMC, 2025) |
| Vaccine-related Adverse Event Cost per Recommended Dosage | $0.20 | (Rite Aid, 2024) |
| Cost of Productivity Loss due to attending vaccination | $46.70 | See section 3.1 |
| Direct medical cost per case hospitalized case 18–49-year-olds | $29,848.86 | (Talbird et al., 2021) |
| Direct medical cost per case hospitalized case 50–59-year-olds | $57,074.73 |  |
| Direct medical cost per case hospitalized case 60–64-year-olds | $57,074.73 |  |
| Direct medical cost per case hospitalized case 65+ year olds | $28,290.06 |  |
| Direct medical cost per case ED case 18–49-year-olds | $443.18 |  |
| Direct medical cost per case ED case 50–59-year-olds | $443.18 |  |
| Direct medical cost per case ED case 60–64-year-olds | $443.18 |  |
| Direct medical cost per case ED case 65+ year olds | $443.18 |  |
| Direct medical cost per case outpatient case 18–49-year-olds | $407.15 | (Kim DeLuca et al., 2023) |
| Direct medical cost per case outpatient case 50–59-year-olds | $407.15 |  |
| Direct medical cost per case outpatient case 50–64-year-olds | $407.15 |  |
| Direct medical cost per case outpatient case 65+ year olds | $781.67 | (Kim DeLuca et al., 2023) |
| Direct medical cost per case NMA case 18–49-year-olds | $0.00 | Assumption |
| Direct medical cost per case NMA case 50–59-year-olds | $0.00 |  |
| Direct medical cost per case NMA case 60–64-year-olds | $0.00 |  |
| Direct medical cost per case NMA case 65+ | $0.00 |  |
| Indirect (productivity) cost per case 18-49 | $586.14 | See section 3.2.3. |
| Indirect (productivity) cost per case 50-59 | $548.80 |  |
| Indirect (productivity) cost per case 60-64 | $548.80 |  |
| Indirect (productivity) cost per case 65+ | $160.90 |  |

5.4 COVID-19

#### 5.4.1 Model description

The COVID-19 model is implemented as a multi-cohort model, tracking annual cohorts from the time of vaccination until age 100 or death. The base case cohort begins at age 65 and runs in annual cycles for 35 years. We model one vaccination course for all individuals aged 65+, as well as one course for risk-based groups over the age of 18. As in other programs, at-risk individuals included those with comorbid conditions (moderate risk) as well as immunocompromised (high risk).

The model follows the general decision tree used across the four programs adapted from Talbird et al. (2021), as shown in Figure 5. While the decision-tree includes ED, we do not generally use this branch as we capture ED (and ICU) incidences, outcomes and costs by adjusting to include them within hospitalization.

Figure 5 Covid-19 decision tree model
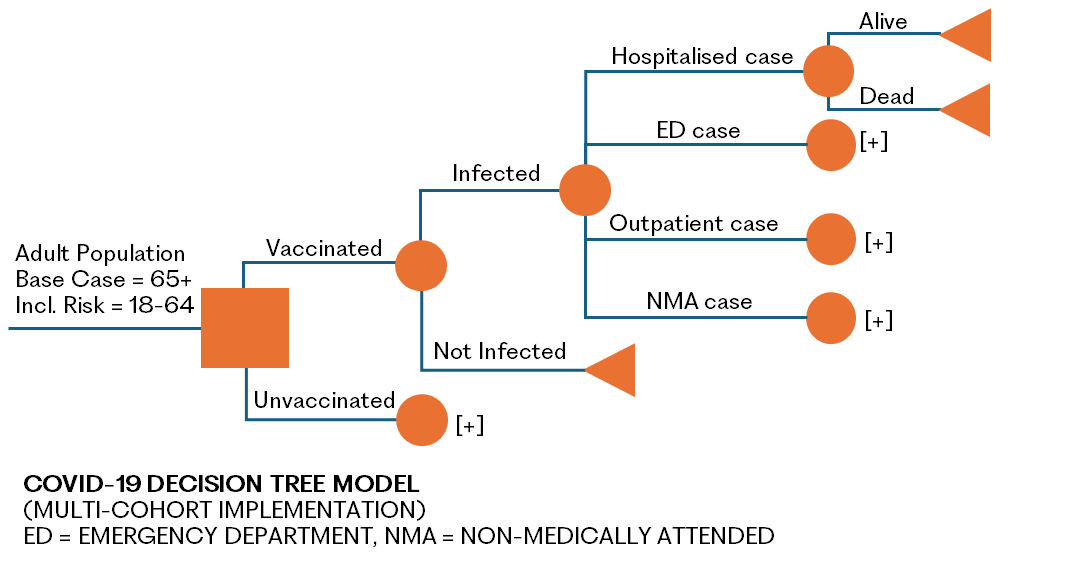


#### 5.4.2 Vaccination program specifications

Full details and sources for all COVID-19 parameters are detailed in Table 4, below.

Table 4 Covid-19 parameters

*Costs are reported in USD2024.*

| **Parameter** | **Value** | **Source** |
| --- | --- | --- |
| Vaccination age (age-based) | 65 | (CDC, 2025i) |
| Vaccination Age (at-risk) | 18 | (CDC, 2025i) |
| Total population cohort size (age-based) | 59,874,349 | (United Nations, Department of Economic and Social Affairs, Population Division, 2024) |
| Total population cohort size (at-risk) | 67,694,587 |  |
| Cohort 18-64 (at-risk) Vaccine Coverage % | 13.80% | (CDC, 2025a; Our World in Data, 2024) |
| Cohort 50-64 (at-risk) Vaccine Coverage % | 23.40% | (CDC, 2025a) |
| Cohort 65-74 Vaccine Coverage % | 46.07% | (CDC, 2025a) |
| Cohort 75+ Vaccine Coverage % | 47.5% | (CDC, 2025a) |
| Vaccine Efficacy against hospitalization/ED (12 month) | 27.04% | Parameter derived from an ongoing trial, currently on file. Available on request. |
| Vaccine Efficacy against hospitalization/ED (6 month) | 42.00% |  |
| Vaccine Efficacy against outpatient (12 month) | 20.88% |  |
| Vaccine Efficacy against outpatient (6 month) | 31.33% |  |
| Vaccine Efficacy against NMA (12 month) | 20.88% |  |
| Vaccine Efficacy against NMA (6 month) | 31.33% |  |
| Incidence per 100,000 non-booster 18–64-year-olds (at-risk) | 8,749 | (CDC, 2023) |
| Incidence per 100,000 non-booster 65–74-year-olds | 29,097 |  |
| Incidence per 100,000 non-booster 75+ year olds | 28,923 |  |
| Hospitalized cases 18–64-year-olds (at-risk) % | 0.83% | (CDC, 2025b) |
| Hospitalized cases 65–74-year-olds % | 2.00% | (CDC, 2025b) |
| Hospitalized cases 75+ year olds % | 5.89% |  |
| Emergency department cases 18–64-year-olds (at-risk) % | 0.00% | Branch not used |
| Emergency department cases 65–74-year-olds % | 0.00% |  |
| Emergency department cases 75+ year olds % | 0.00% |  |
| Outpatient cases 18–64-year-olds (at-risk) | 51.10% | (Sah et al., 2021; Accorsi et al., 2022) |
| Outpatient cases 65–74-year-olds % | 51.10% |  |
| Outpatient cases 75+ year olds % | 51.10% |  |
| Nonmedically attended cases of 18–64-year-olds (at-risk) | 48.07% | Calculated based on 100% - outpatient case % - hospitalized case % |
| Nonmedically attended cases 65–74-year-olds % | 46.90% |  |
| Nonmedically attended cases 75+ year olds % | 43.01% |  |
| Deaths from hospitalized cases 18–64-year-olds (at-risk) | 1.06% | (Adams, Katz and Grandpre, 2020; Kompaniyets, 2021; Wisk and Sharma, 2025) |
| Deaths from hospitalized cases for 65–74-year-olds % | 2.55% | (Yehoshua et al., 2024) |
| Deaths from hospitalized cases for 75+ year olds % | 3.41% |  |
| Deaths from ED cases for 18-64 (at-risk) year olds % | 0.00% | Assumption |
| Deaths from ED cases for 65–74-year-olds % | 0.00% |  |
| Deaths from ED cases for 75+ year olds % | 0.00% |  |
| Deaths from outpatient cases for 18-64 (at-risk) year olds % | 0.00% |  |
| Deaths from outpatient cases for 65–74-year-olds % | 0.00% |  |
| Deaths from outpatient cases for 75+ year olds % | 0.00% |  |
| Deaths from NMA cases for 18-64 (at-risk) year olds % | 0.00% |  |
| Deaths from NMA cases for 65–74-year-olds % | 0.00% |  |
| Deaths from NMA cases for 75+ year olds % | 0.00% |  |
| Vaccine Acquisition Cost per Recommended Dosage | $136.75 | (CDC, 2025c) |
| Vaccine Administration Cost per Recommended Dosage | $20.54 | (CMC, 2025) |
| Vaccine-related Adverse Event Cost per Recommended Dosage | $0.20 | (Rite Aid, 2024) |
| Cost of Productivity Loss due to attending vaccination (Age-Based) | $46.70 | See section 3.1 |
| Cost of Productivity Loss due to attending vaccination (Risk-Included) | $0.00 | Assumption |
| Direct medical cost per case hospitalized case 18-64 (at-risk) year olds | $17,028.50 | (Yehoshua et al., 2024) |
| Direct medical cost per case hospitalized case 65–74-year-olds | $18,413.88 |  |
| Direct medical cost per case hospitalized case 75+ year olds | $16,069.24 |  |
| Direct medical cost per case ED case 18-64 (at-risk) year olds | $0.00 | Branch not used |
| Direct medical cost per case ED case 65–74-year-olds | $0.00 |  |
| Direct medical cost per case ED case 75+ year olds | $0.00 |  |
| Direct medical cost per case outpatient case 18-64 (at-risk) year olds | $793.00 | (Yehoshua et al., 2024) |
| Direct medical cost per case outpatient case 65–74-year-olds | $793.00 |  |
| Direct medical cost per case outpatient case 75+ year olds | $793.00 |  |
| Direct medical cost per case NMA case 18-64 (at-risk) year olds | $0.00 | Assumption |
| Direct medical cost per case NMA case 65–74-year-olds | $0.00 |  |
| Direct medical cost per case NMA case 75+ year olds | $0.00 |  |
| Indirect (productivity) cost per hospitalized case 18-49 | $1,127.19 | See section 3.2.3 |
| Indirect (productivity) cost per hospitalized case 50-64 | $1,055.39 | See section 3.2.3. |
| Indirect (productivity) cost per hospitalized case 65+ | $309.41 |  |
| Indirect (productivity) cost per ED (ICU) case 18-49 | $811.58 |  |
| Indirect (productivity) cost per ED (ICU) case 50-64 | $759.88 |  |
| Indirect (productivity) cost per ED (ICU) case 65+ | $222.78 |  |
| Indirect (productivity) cost per outpatient case 18-49 | $811.58 |  |
| Indirect (productivity) cost per outpatient case 50-64 | $759.88 |  |
| Indirect (productivity) cost per outpatient case 65+ | $222.78 |  |
| Indirect (productivity) cost per NMA case 18-49 | $275.03 |  |
| Indirect (productivity) cost per NMA case 50-64 | $257.52 |  |
| Indirect (productivity) cost per NMA case 65+ | $75.50 |  |

5.5 Comparative contextualization of epidemiological inputs

The core epidemiological inputs used in our models, including incidence, outcome probability, and vaccine effectiveness, are largely drawn from the most recent peer-reviewed evidence base that also often informs the cost-effectiveness analyses (CEA) used in ACIP policy deliberation, specifically those by Hutton et al. (2024) for RSV, Stoecker (2024) for PD, and Prosser et al. (2025) or the most recent CDC data for influenza and COVID-19.

Because our analysis is a societal-perspective BCA rather than a payer-perspective CEA, certain structural differences exist, most notably the inclusion of non-medically attended illness and the use of VSL/VSLY-based mortality valuation rather than quality adjusted life years (QALYs). Across all four diseases, differences in inputs compared to ACIP models, reflects the broader scope of a societal BCA, the availability of more recent data, or conservative modelling choices. Below we note key areas of alignment and difference by disease area.

For Flu and COVID-19, our inputs are closely aligned with the ACIP evidence base. COVID-19 incidence, hospitalisation rates, and case fatality rates are derived from CDC burden surveillance data, the same databases are used by Prosser et al. (2025). Flu VE estimates reflect CDC-reported seasonal averages, while our COVID-19 VE estimates against hospitalisation (42% at 6 months; 27% at 12 months), obtained from recent trial data are broadly consistent with the observational effectiveness data (Link-Gelles et al., 2025) that also underpin used by Prosser, which use seasonality-adjusted VE against symptomatic illness/hospitalisation of 34.7% and VE against ICU/death of 45.1%VE; importantly, the two models parameterise VE differently.

For PD, core inputs share the same evidence base as the ACIP economic assessment of PCV21 in US adults (Stoecker, 2024). Incidence estimates are informed by Talbird et al. (2021), drawing on overlapping surveillance data used by Stoecker (2024). Furthermore, VE against IPD (75%) is drawn from the CAPiTA trial (Bonten et al., 2015) in both analyses, however they parameterise VE against NBPP differently, they are in a similar order of magnitude (66.7%, 40.3%, 15.0% from Suaya et al. (2018) compared to our 45% (Bonten et al., 2015). Case fatality rates are broadly comparable; our age-based estimates (10.7% for 50-64 years and 14.9% for 65+) sit between Stoecker's general and CMC strata (8.73% for 50-64 years up to 17.3% for 85+), and respective values for the risk populations aged 18-49 are 6.22% and 7.24%. Three structural differences are noted. First, Stoecker stratifies incidence, CFR, and VE by risk group (general population, chronic medical conditions - CMC, immunocompromised - IC), whereas our model considers a single at-risk population encompassing both CMC and IC individuals, and we also use broader age bands reflecting a population-average approach. Second, Stoecker models serotype 3 VE separately at a lower level (9%-26% across risk groups for IPD; 5.2%-15.6% for NBPP), whereas we apply a blended VE across all vaccine-type serotypes. Third, waning assumptions also differ because we follow the more recent evidence from Gourzoulidis et al. (2023) while Stoecker uses Patterson et al. (2016) and van Werkhoven et al. (2015), which differ slightly in trajectory although cover the same total duration of 15 years.

For RSV, differences arise between Averin et al., (2024) which is the source of our parameters and Hutton et al. (2024). Our incidence estimates of 462 per 100,000 are markedly larger than the 303 per 100,000 used by Hutton et al. (2024), which can mostly be attributed to the value used to adjust for under-reporting. Both sources adjust for under-reporting with Hutton using a 1.5× multiplier (Kujawski et al., 2022) and Averin using 1.95x from more recently published data (Onwuchekwa et al., 2023; Ramirez et al., 2023). Our hospitalised case fatality rate (9.8%) is also higher than that used in Hutton's model (5.7%), meanwhile, VE estimates are closely aligned. Our Year 1 weighted average VE against hospitalisation of 82.5%, calculated using vaccine effectiveness data from Tartof et al., (2025b; a), is comparable to Hutton's real-world observational estimate (84.6% at months 0-7). Furthermore, our VE against outpatient illness is 62.7%, derived from Walsh et al., (2023) and Hutton's estimate is 65.2%. The most impactful variance is in the assumed duration of protection. Hutton assumes protection wanes linearly to zero by 24 months, based on clinical trial follow-up available at the time of analysis. Our model assumes protection against hospitalisation persists over 7 years, informed by more recently available real-world evidence from Kaiser Permanente & Tartof et al., (2025b; a).

## 6. Detailed results

### 6.1. Full program value (age-based recommendations)

Table 5 presents the full set of aggregated discounted results for the current CDC age-based recommendations across 1-, 7-, 15-year, and lifetime horizons. The lifetime horizon represents the maximum model runtime of 82 years, as the youngest eligible cohort enters at age 18. Each cohort is followed from its eligibility age to age 100 or death; consequently, older cohorts contribute fewer years of follow-up than younger ones (e.g., a cohort vaccinated at age 75 contributes up to 25 years). The results in Table 5 extend the results reported in the main text by including total program costs, a breakdown of monetised benefits into mortality, medical, and productivity components, the tangible offset, and mean net benefits per vaccination course.

Under age-based recommendations, total program costs over 15 years amount to approximately $205 billion. Monetised mortality benefits account for the majority of total benefits, at $2.4 trillion (VSL) or $839 billion (VSLY) by Year 15. Medical cost savings contribute $87 billion and averted productivity losses a further $25 billion over the same period. The tangible offset, the share of total benefits attributable to medical and productivity gains independent of mortality valuation, rises from 22% at Year 1 to 54% at Year 15, reflecting the growing contribution of averted healthcare utilisation over time. Mean net benefits per vaccination course reach $12,171 (VSL) or $3,029 (VSLY) at Year 15.

Table 5: aggregated discounted results for age-based recommendations

|  | **Year 1** | **Year 7** | **Year 15** | **Lifetime** |
| --- | --- | --- | --- | --- |
| **BCR (VSL)** | 4.94 | 11.33 | 12.50 | 14.82 |
| **BCR (VSLY)** | 1.91 | 4.31 | 4.65 | 5.18 |
| **NMB (VSL)** | $179,965,597,697 | $1,301,330,384,980 | $2,351,282,570,097 | $4,797,023,876,927 |
| **NMB (VSLY)** | $41,595,119,942 | $417,539,633,101 | $745,743,607,631 | $1,450,607,692,449 |
| **ROI (VSL)** | 540% | 1363% | 1505% | 1785% |
| **ROI (VSLY)** | 91% | 331% | 365% | 418% |
| **Hospitalised cases prevented** | 228,993 | 1,437,703 | 2,524,940 | 4,281,846 |
| **Hospital bed-days freed up** | 1,398,241 | 8,657,367 | 15,106,956 | 25,495,105 |
| **Outpatient cases prevented** | 2,772,335 | 16,883,909 | 30,722,185 | 57,455,414 |
| **NMA cases prevented** | 2,993,289 | 17,705,118 | 33,009,697 | 58,360,155 |
| **Deaths averted** | 16,074 | 101,784 | 182,419 | 369,001 |
| **Total Program Costs** | $45,699,602,202 | $126,003,579,677 | $204,519,534,367 | $347,013,506,808 |
| **Total Monetised Benefits (VSL)** | $225,665,199,899 | $1,427,333,964,657 | $2,555,802,104,463 | $5,144,037,383,734 |
| **Total Monetised Benefits (VSLY)** | $87,294,722,144 | $543,543,212,778 | $950,263,141,998 | $1,797,621,199,257 |
| **Monetised Mortality Benefit (VSL)** | $215,388,827,812 | $1,363,908,558,316 | $2,444,407,904,579 | $4,944,610,638,844 |
| **Monetised Mortality Benefit (VSLY)** | $77,018,350,058 | $480,117,806,437 | $838,868,942,113 | $1,598,194,454,366 |
| **Morbidity-related Medical Benefit** | $8,071,847,459 | $49,952,738,473 | $86,895,698,979 | $156,070,529,581 |
| **Morbidity-related Productivity Benefit** | $2,204,524,627 | $13,472,667,868 | $24,498,500,906 | $43,356,215,309 |
| **Tangible Offset** | 22% | 50% | 54% | 57% |
| **Mean NBs per vaccination course (VSL)** | $1759.3 | $8475 | $12,171 | $18,094 |
| **Mean NBs per vaccination course (VSLY)** | $327.5 | $2058 | $3,029 | $4,606 |

### 6.2. Full program value (age- and risk-based recommendations)

Table 6 presents the corresponding results when risk-based recommendations are included alongside age-based eligibility. Total program costs over 15 years increase to approximately $246 billion, reflecting the larger eligible population. Including at-risk populations increases total monetised benefits at Year 15 to $2.8 trillion (VSL) or $1.1 trillion (VSLY), with monetised mortality benefits of $2.6 trillion (VSL) or $944 billion (VSLY). Medical cost savings reach $100 billion and averted productivity losses $27 billion. The tangible offset at Year 15 is 52%, slightly lower than under age-based recommendations alone, as the inclusion of younger at-risk adults increases the relative contribution of mortality valuation. Mean net benefits per vaccination course are $10,278 (VSL) or $3,172 (VSLY) at Year 15, lower per course than the age-based analysis due to the inclusion of lower-risk younger cohorts, but generating higher aggregate net benefits overall.

Table 6: AGGREGATED DISCOUNTED RESULTS FOR AGE- AND RISK-BASED RECOMMENDATIONS

|  | **Year 1** | **Year 7** | **Year 15** | **Lifetime** |
| --- | --- | --- | --- | --- |
| **BCR (VSL)** | 4.71 | 10.62 | 11.19 | 12.81 |
| **BCR (VSLY)** | 1.97 | 4.29 | 4.36 | 4.54 |
| **NMB (VSL)** | $200,378,887,497 | $1,420,367,779,041 | $2,507,940,276,447 | $5,245,098,566,055 |
| **NMB (VSLY)** | $52,601,582,091 | $485,932,104,296 | $825,520,369,349 | $1,574,376,271,403 |
| **ROI (VSL)** | 522% | 1291% | 1352% | 1529% |
| **ROI (VSLY)** | 97% | 329% | 336% | 354% |
| **Hospitalised cases prevented** | 267,060 | 1,631,511 | 2,850,734 | 5,365,906 |
| **Hospital bed-days freed up** | 1,676,329 | 10,009,693 | 17,249,128 | 32,186,875 |
| **Outpatient cases prevented** | 3,075,561 | 18,528,404 | 34,656,654 | 68,860,864 |
| **NMA cases prevented** | 3,398,063 | 19,301,223 | 37,270,237 | 68,608,635 |
| **Deaths averted** | 18,050 | 111,522 | 196,025 | 406,916 |
| **Total Program Costs** | $54,010,678,237 | $147,638,351,866 | $246,009,269,011 | $444,120,488,399 |
| **Total Monetised Benefits (VSL)** | $254,389,565,733 | $1,568,006,130,907 | $2,753,949,545,458 | $5,689,219,054,455 |
| **Total Monetised Benefits (VSLY)** | $106,612,260,327 | $633,570,456,162 | $1,071,529,638,360 | $2,018,496,759,802 |
| **Monetised Mortality Benefit (VSL)** | $241,871,107,133 | $1,494,392,622,118 | $2,626,740,720,888 | $5,452,672,236,384 |
| **Monetised Mortality Benefit (VSLY)** | $94,093,801,727 | $559,956,947,372 | $944,320,813,790 | $1,781,949,941,731 |
| **Morbidity-related Medical Benefit** | $9,997,474,714 | $58,675,134,139 | $100,146,888,443 | $187,779,523,426 |
| **Morbidity-related Productivity Benefit** | $2,520,983,887 | $14,938,374,651 | $27,061,936,127 | $48,767,294,645 |
| **Tangible Offset** | 23% | 50% | 52% | 53% |
| **Mean NBs per vaccination course (VSL)** | $1272.3 | $6951 | $10,278 | $17,732 |
| **Mean NBs per vaccination course (VSLY)** | $355.3 | $2266 | $3,172 | $4,996 |

### 6.3. Complementary analysis: net present value (NPV) associated with one year of vaccination activity

Our primary analyses above estimate the full program value of current CDC vaccination recommendations by modelling all eligible cohorts over the duration of vaccine protection. This captures the total societal value of the programs over time. As a complementary analysis, we estimate the net present value (NPV) value of one year of vaccination activity, which represents the incremental societal return from one year's vaccination activity. This addresses the differing administration schedules across the four programs: influenza and COVID-19 vaccines are recommended annually, while PD and RSV vaccines are single-administration with longer durations of protection.

For PD and RSV, the complementary analysis of one year of vaccination activity comprises individuals newly reaching the age-based eligibility threshold (age 50 for PD; age 75 for RSV), plus eligible at-risk adults in younger age groups. Year 1 vaccination costs are included, with downstream benefits tracked over the full duration of protection (15 years for PD; 7 years for RSV). For influenza and COVID-19, the modelled cohorts comprise individuals at and above the age-based eligibility (age 18 for influenza; age 65 for COVID-19), plus eligible at-risk adults, with costs and benefits captured within one year. Where available, uptake rates are used in place of cumulative coverage rates to reflect the annual rate of newly vaccinated individuals. For PD, observed uptake rates are available and are used: 17.95% for age-based; 4.7% for at-risk adults (Averin et al., 2025b). For RSV, as a more recently introduced program, established uptake data are limited; the coverage rates used in the main analysis are therefore retained as a proxy for uptake.

As shown in Table 7, the NPV analysis of one year of vaccination activity yields a BCR of 12:1 (VSL) or 5.6:1 (VSLY), with net benefits of $241 billion (VSL) or $101 billion (VSLY) from approximately $22 billion in annual vaccination costs. This analysis demonstrates that even when framed as an incremental annual investment, adult respiratory vaccination programs generate substantial positive societal returns.

The two approaches are complementary. The full program value analysis answers the question of what current CDC recommendations are worth to society in aggregate. The analysis of one year of vaccination activity provides a measure of the incremental return on each year's vaccination activity that is more directly comparable to annual budgetary decisions. We note that this annual NPV approach is best suited to established steady-state programs; for PD and RSV, which have not yet reached steady state, the full program value analysis better reflects the near-term reality that catch-up cohorts above the eligibility threshold are still being vaccinated.

| Table 7: AGGREGATED DISCOUNTED RESULTS FOR One year of Vaccination activity | **NPV** |
| --- | --- |
| **BCR (VSL)** | **12.00** |
| **BCR (VSLY)** | **5.60** |
| **NMB (VSL)** | **$240,509,205,753** |
| **NMB (VSLY)** | **$100,619,426,843** |
| **ROI (VSL)** | 1528% |
| **ROI (VSLY)** | 460% |
| **Hospitalised cases prevented** | 294,473 |
| **Hospital bed-days freed up** | 1,974,645 |
| **Outpatient cases prevented** | 3,105,994 |
| **NMA cases prevented** | 3,380,468 |
| **Deaths averted** | 18,502 |
| **Total Program Costs** | $21,868,977,464 |
| **Total Monetised Benefits (VSL)** | $262,378,183,217 |
| **Total Monetised Benefits (VSLY)** | $122,488,404,307 |
| **Monetised Mortality Benefit (VSL)** | $247,923,743,812 |
| **Monetised Mortality Benefit (VSLY)** | $108,033,964,902 |
| **Morbidity-related Medical Benefit** | $11,728,811,400 |
| **Morbidity-related Productivity Benefit** | $2,725,628,005 |
| **Tangible Offset** | 66% |
| **Mean NBs per vaccination course (VSL)** | $4,067 |
| **Mean NBs per vaccination course (VSLY)** | $2,337 |

## 7. Sensitivity analyses & robustness checks

### 7.1. One-way sensitivity analyses results

Below we present the top 25 most impactful parameters among all programs against the aggregated BCR at year 15, across all four programs for both VSL valuation (Figure 6) and VSLY (Figure 7).

Due to greater eligibility in recommendations, and therefore larger resultant populations for the flu and COVID programs, these parameters have the most impact on the aggregated BCR. We note that this does not allow for any direct comparisons of the BCR between programs, as the underlying population weights differ strongly by individual TA.

Figure 6 one-way sensitivity analysis against aggregated 15-year bcr (VSL)


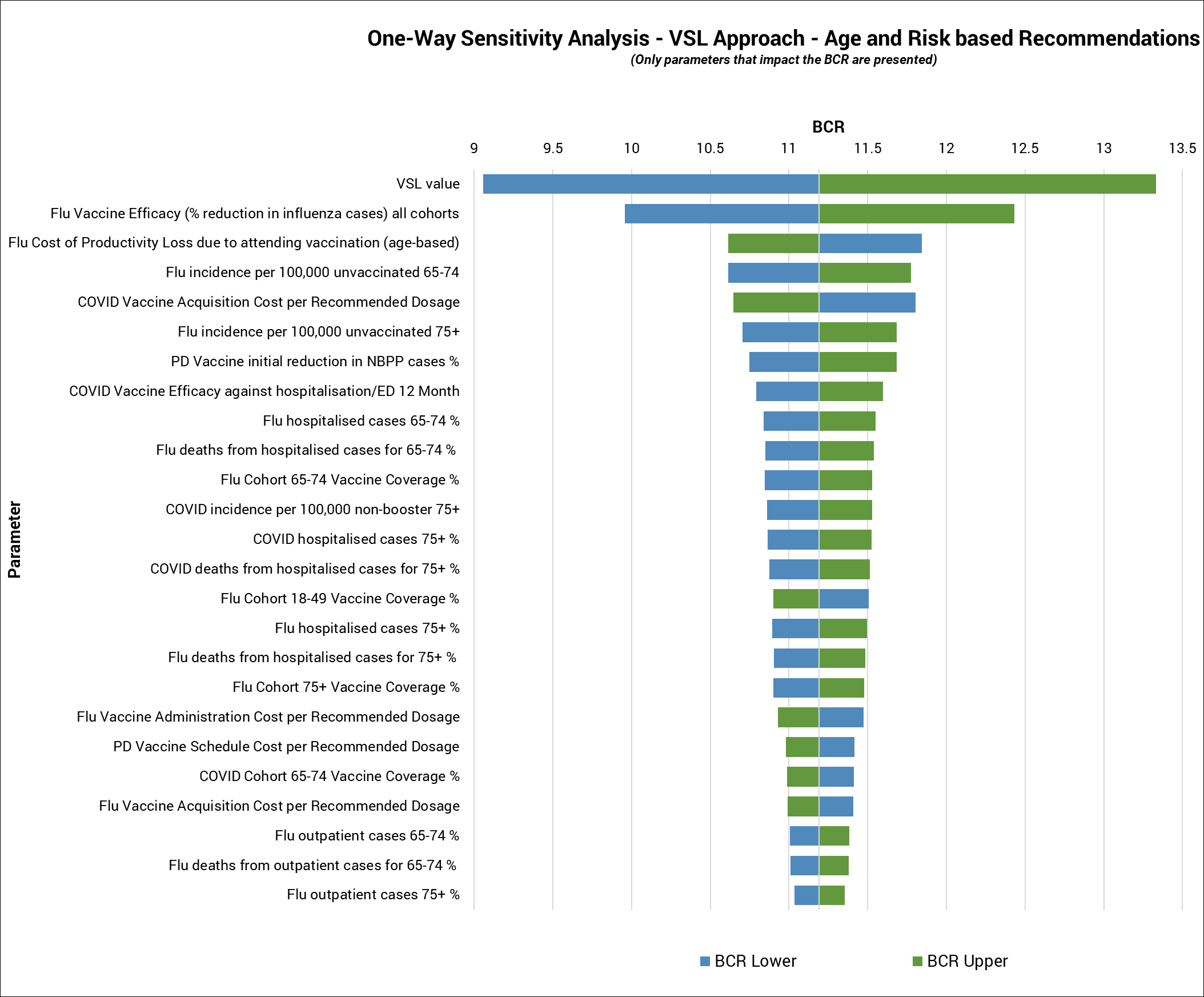


Figure 7: ONE-WAY SENSITIVITY ANALYSIS AGAINST AGGREGATED 15-YEAR BCR (VSLY)


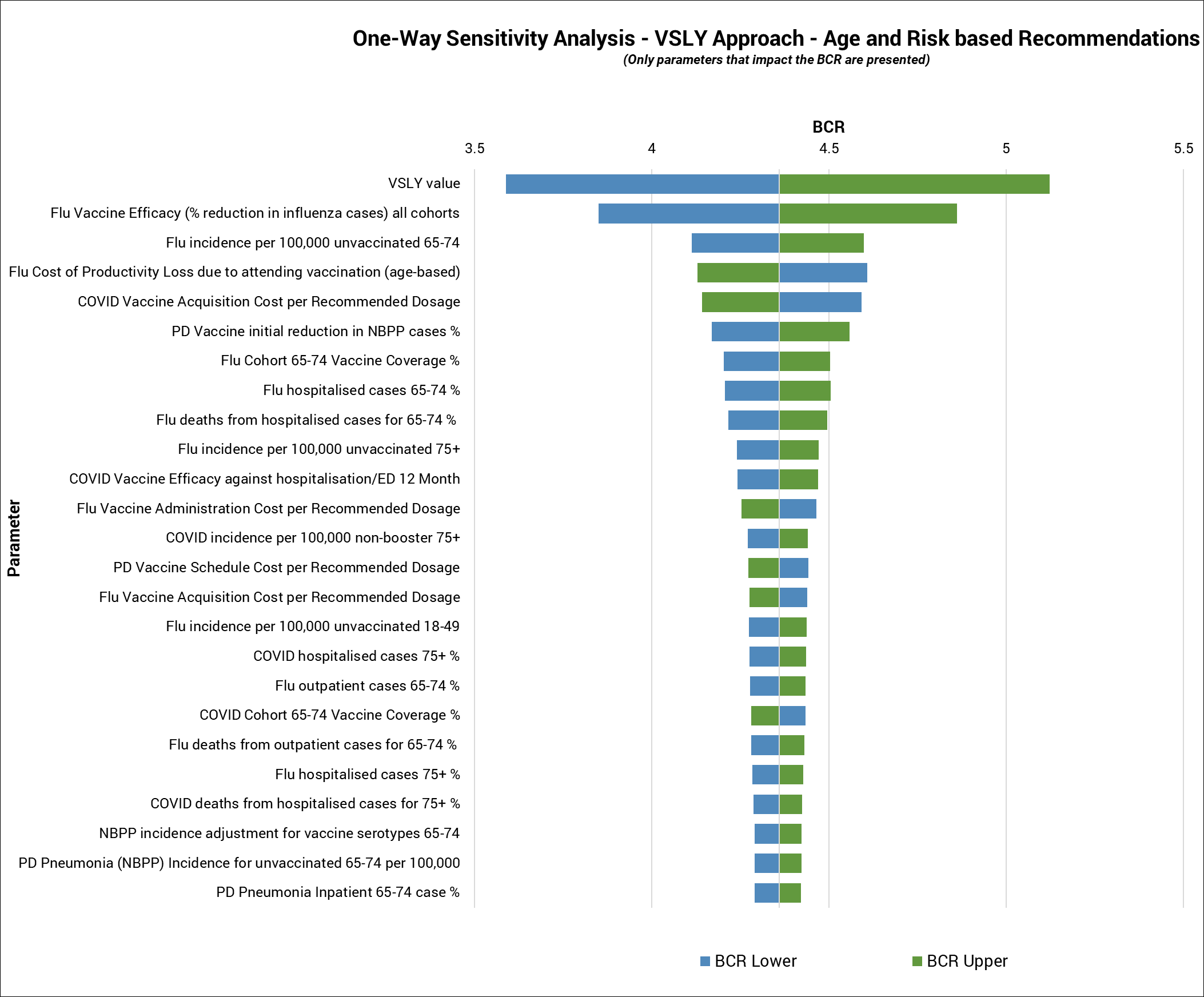


### 7.2. Human capital valuation of mortality

In the base case, mortality risk reduction is valued using the VSL and VSLY from the US Department of Health and Human Services VSL estimate (Kearsley, 2025). VSLY is adjusted for remaining life expectancy at the age of mortality. As a robustness check, we replace the VSLY with age-specific annual productivity estimates from Grosse et al., (2019), inflated to 2024 US dollars (~$73,072 per year). This human capital approach (HCA) captures the present value of averted market and non-market productivity losses from premature death but excludes the intangible elements of society's willingness to pay for mortality risk reduction that are reflected in the VSL and VSLY.

As shown in Table 8, replacing VSLY with HCA substantially reduces estimated net benefits, as expected, given that the HCA per-year value is approximately 80% lower than the VSLY. At Year 1, the BCR falls below 1 (0.59), reflecting high upfront vaccination costs for PD and RSV. However, from Year 7 onwards, benefits exceed costs under HCA (BCR 1.28), and by Year 15, the programs generate $76.5 billion in net benefits with a BCR of 1.31. These results confirm that adult respiratory vaccination programs generate positive societal returns even under a conservative valuation that excludes intangible welfare gains from prevented mortality.

Below we also present the top 25 most impactful parameters among all programs against the aggregated BCR at year 15, across all four programs when using human capital estimates from Grosse et al., (2019), to value mortality (Figure 8).

Table 8: AGGREGATED DISCOUNTED RESULTS FOR AGE- AND RISK-BASED RECOMMENDATIONS, Human capital valuation of mortality

|  | **Year 1** | **Year 7** | **Year 15** | **Lifetime** |
| --- | --- | --- | --- | --- |
| **BCR** | 0.59 | 1.28 | 1.31 | 1.36 |
| **NMB** | -$22,035,072,063 | $41,765,620,903 | $76,470,541,457 | 160,906,105,678 |
| **ROI** | -41% | 28% | 31% | 36% |
| **Hospitalised cases prevented** | 267,060 | 1,631,511 | 2,850,734 | 5,365,906 |
| **Hospital bed-days freed up** | 1,676,329 | 10,009,693 | 17,249,128 | 32,186,875 |
| **Outpatient cases prevented** | 3,075,561 | 18,528,404 | 34,656,654 | 68,860,864 |
| **NMA cases prevented** | 3,398,063 | 19,301,223 | 37,270,237 | 68,608,635 |
| **Deaths averted** | 18,050 | 111,522 | 196,025 | 406,916 |
| **Total Program Costs** | $54,010,678,237 | $147,638,351,866 | $246,009,269,011 | $444,120,488,399 |
| **Total Monetised Benefits** | $31,975,606,173 | $189,403,972,769 | $322,479,810,469 | $605,026,594,078 |
| **Monetised Mortality Benefit** | $19,457,147,573 | $115,790,463,979 | $195,270,985,898 | $368,479,776,007 |
| **Morbidity-related Medical Benefit** | $9,997,474,714 | $58,675,134,139 | $100,146,888,443 | $187,779,523,426 |
| **Morbidity-related Productivity Benefit** | $2,520,983,887 | $14,938,374,651 | $27,061,936,127 | $48,767,294,645 |
| **Tangible Offset** | 23% | 50% | 52% | 53% |
| **Mean NBs per vaccination course** | -$65.5 | $264.0 | $298.3 | $399.5 |


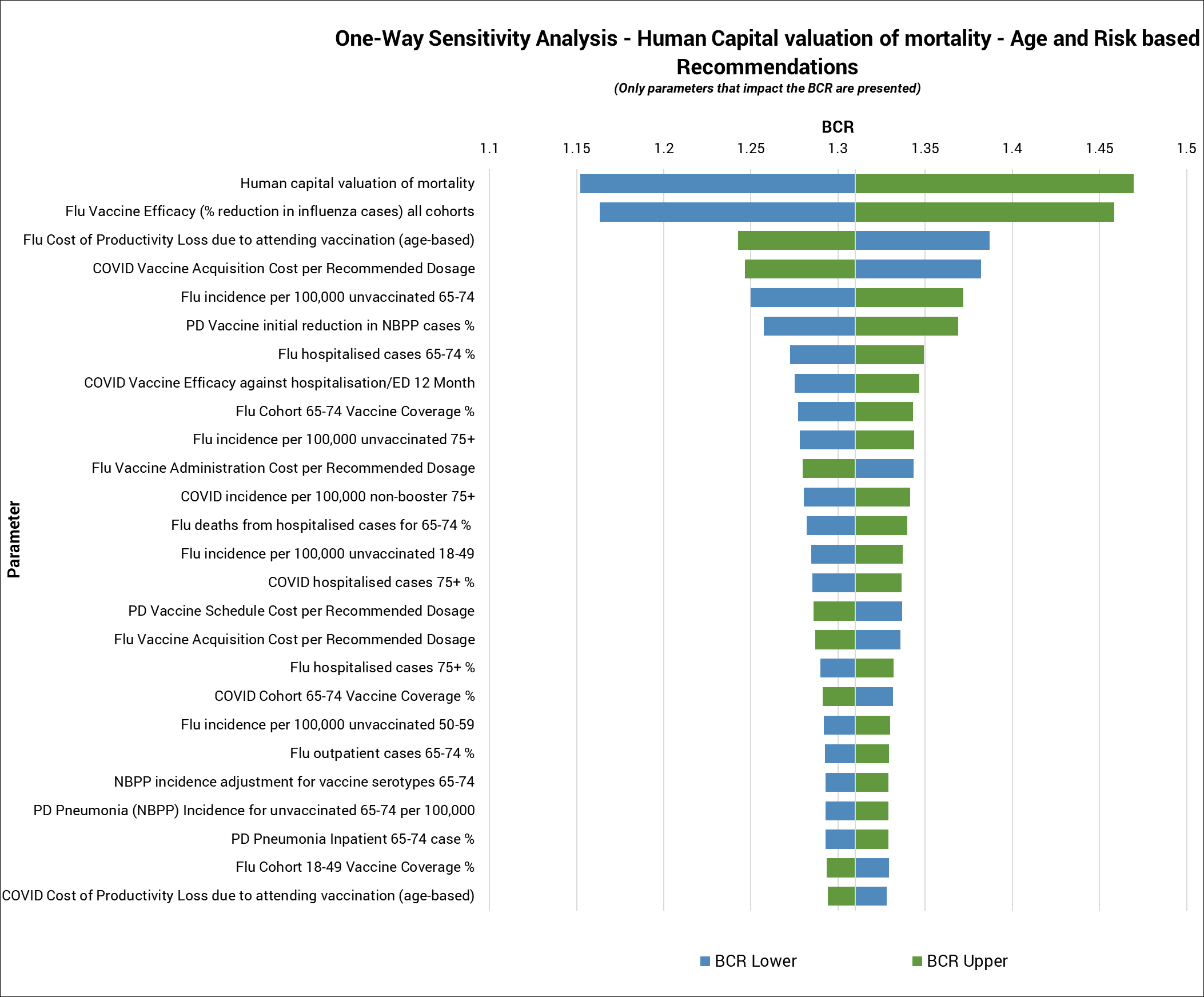


Figure 8:ONE-WAY SENSITIVITY ANALYSIS AGAINST AGGREGATED 15-YEAR BCR (HUMAN CAPITAL)

### 7.3. Human capital valuation of productivity

In the base case analysis, productivity losses are valued using mean annual wages, applied to employed individuals by age group. As a robustness check, we adopt the human capital approach (HCA) estimates from Grosse et al., (2019), which capture both market productivity (gross earnings adjusted for employer-paid benefits) and non-market productivity (imputed value of household, caring and volunteer services), averaged across the non-institutionalized population. The 2016 mean annual productivity of $57,324 was inflated to 2024 US dollars (~$73,072) and applied to value both productivity losses from illness and the time cost of attending vaccination.

As shown in Table 9, this alternative valuation has a negligible effect on results. BCRs and net benefits remain consistent with the base case, confirming that results are robust to alternative productivity valuation methods.

Table 9: AGGREGATED DISCOUNTED RESULTS FOR AGE- AND RISK-BASED RECOMMENDATIONS, Human capital valuation of PRODUCTIVITY

|  | **Year 1** | **Year 7** | **Year 15** | **Lifetime** |
| --- | --- | --- | --- | --- |
| **BCR (VSL)** | 4.80 | 10.93 | 11.55 | 13.26 |
| **BCR (VSLY)** | 2.01 | 4.41 | 4.49 | 4.70 |
| **NMB (VSL)** | $201,204,948,105 | $1,423,336,650,699 | $2,513,341,010,862 | $5,256,226,364,424 |
| **NMB (VSLY)** | $53,427,642,699 | $488,900,975,954 | $830,921,103,764 | $1,585,504,069,772 |
| **ROI (VSL)** | 534% | 1333% | 1399% | 1588% |
| **ROI (VSLY)** | 101% | 341% | 349% | 370% |
| **Hospitalised cases prevented** | 267,060 | 1,631,511 | 2,850,734 | 5,365,906 |
| **Hospital bed-days freed up** | 1,676,329 | 10,009,693 | 17,249,128 | 32,186,875 |
| **Outpatient cases prevented** | 3,075,561 | 18,528,404 | 34,656,654 | 68,860,864 |
| **NMA cases prevented** | 3,398,063 | 19,301,223 | 37,270,237 | 68,608,635 |
| **Deaths averted** | 18,050 | 111,522 | 196,025 | 406,916 |
| **Total Program Costs** | $52,963,000,808 | $143,356,264,718 | $238,229,550,634 | $428,705,612,739 |
| **Total Monetised Benefits (VSL)** | $254,167,948,913 | $1,566,692,915,417 | $2,751,570,561,496 | $5,684,931,977,164 |
| **Total Monetised Benefits (VSLY)** | $106,390,643,507 | $632,257,240,672 | $1,069,150,654,398 | $2,014,209,682,512 |
| **Monetised Mortality Benefit (VSL)** | $241,871,107,133 | $1,494,392,622,118 | $2,626,740,720,888 | $5,452,672,236,384 |
| **Monetised Mortality Benefit (VSLY)** | $94,093,801,727 | $559,956,947,372 | $944,320,813,790 | $1,781,949,941,731 |
| **Morbidity-related Medical Benefit** | $9,997,474,714 | $58,675,134,139 | $100,146,888,443 | $187,779,523,426 |
| **Morbidity-related Productivity Benefit** | $2,299,367,066 | $13,625,159,161 | $24,682,952,165 | $44,480,217,354 |
| **Tangible Offset** | 23% | 50% | 52% | 54% |
| **Mean NBs per vaccination course (VSL)** | $1275.2 | $6960 | $10,293 | $17,763 |
| **Mean NBs per vaccination course (VSLY)** | $358.1 | $2274 | $3,188 | $5,028 |

## 8. References

Accorsi, E.K., Britton, A., Fleming-Dutra, K.E., Smith, Z.R., Shang, N., Derado, G., Miller, J., Schrag, S.J. and Verani, J.R., 2022. Association Between 3 Doses of mRNA COVID-19 Vaccine and Symptomatic Infection Caused by the SARS-CoV-2 Omicron and Delta Variants. *JAMA*, 327(7), pp.639–651. 10.1001/jama.2022.0470.

Adams, M.L., Katz, D.L. and Grandpre, J., 2020. Population-Based Estimates of Chronic Conditions Affecting Risk for Complications from Coronavirus Disease, United States - Volume 26, Number 8—August 2020 - Emerging Infectious Diseases journal - CDC. [online] 10.3201/eid2608.200679.

Averin, A., Huebbe ,Bennet, Atwood ,Mark, Bayer ,Lea J, Lade ,Caroline, von Eiff ,Christof and and Sato, R., 2025a. Cost-effectiveness of bivalent respiratory syncytial virus prefusion F vaccine for prevention of respiratory syncytial virus among older adults in Germany. *Expert Review of Vaccines*, 24(1), pp.1–10. 10.1080/14760584.2024.2436183.

Averin, A., Sato, R., Begier, E., Gessner, B.D., Snow, V., Cane, A., Quinn, E., Atwood, M., Kijauskaite, G. and Weycker, D., 2024. Annual public health and economic burden of medically attended respiratory syncytial virus illnesses among US adults. *Vaccine*, 42(26), p.126323. 10.1016/j.vaccine.2024.126323.

Averin, A., Vietri, J., Mohs, A.A., Willis, S.J., Lonshteyn, A. and Weycker, D., 2025b. Uptake of Pneumococcal Vaccines Among U.S. Adults After 2022 Update to Recommendations. *AJPM focus*, 4(5), p.100384. 10.1016/j.focus.2025.100384.

Bonten, M.J.M., Huijts, S.M., Bolkenbaas, M., Webber, C., Patterson, S., Gault, S., Werkhoven, C.H. van, Deursen, A.M.M. van, Sanders, E.A.M., Verheij, T.J.M., Patton, M., McDonough, A., Moradoghli-Haftvani, A., Smith, H., Mellelieu, T., Pride, M.W., Crowther, G., Schmoele-Thoma, B., Scott, D.A., Jansen, K.U., Lobatto, R., Oosterman, B., Visser, N., Caspers, E., Smorenburg, A., Emini, E.A., Gruber, W.C. and Grobbee, D.E., 2015. Polysaccharide Conjugate Vaccine against Pneumococcal Pneumonia in Adults. *New England Journal of Medicine*, 372(12), pp.1114–1125. 10.1056/NEJMoa1408544.

CDC, 2023. *Rates of COVID-19 Cases or Deaths by Age Group and Updated (Bivalent) Booster Status | Data | Centers for Disease Control and Prevention*. [online] Available at: https://data.cdc.gov/Public-Health-Surveillance/Rates-of-COVID-19-Cases-or-Deaths-by-Age-Group-and/54ys-qyzm/about_data [Accessed 21 Nov. 2025].

CDC, 2024a. *Current Epidemiology of Pneumococcal Disease among Adults, United States*.

CDC, 2024b. *Flu Vaccination Coverage, United States, 2021–22 Influenza Season*. [online] FluVaxView. Available at: https://www.cdc.gov/fluvaxview/coverage-by-season/2021-2022.html [Accessed 21 Nov. 2025].

CDC, 2024c. *Past Seasons’ Vaccine Effectiveness Estimates*. [online] Flu Vaccines Work. Available at: https://www.cdc.gov/flu-vaccines-work/php/effectiveness-studies/past-seasons-estimates.html [Accessed 6 May 2025].

CDC, 2025a. *COVID-19 Vaccination Coverage and Intent for Vaccination, Adults 18 Years and Older, United States*. [online] COVIDVaxView. Available at: https://www.cdc.gov/covidvaxview/weekly-dashboard/adult-vaccination-coverage.html [Accessed 21 Nov. 2025].

CDC, 2025b. *COVID-NET*. [online] Available at: https://www.cdc.gov/covid/php/covid-net/index.html [Accessed 24 Oct. 2025].

CDC, 2025c. *Current CDC Vaccine Price List*. [online] Vaccines for Children Program. Available at: https://www.cdc.gov/vaccines-for-children/php/awardees/current-cdc-vaccine-price-list.html [Accessed 24 Oct. 2025].

CDC, 2025d. *Flu Disease Burden: Past Seasons*. [online] Flu Burden. Available at: https://www.cdc.gov/flu-burden/php/data-vis/past-seasons.html [Accessed 21 Nov. 2025].

CDC, 2025e. *Past Seasons’ Vaccine Effectiveness Estimates*. [online] Flu Vaccines Work. Available at: https://www.cdc.gov/flu-vaccines-work/php/effectiveness-studies/past-seasons-estimates.html [Accessed 22 Oct. 2025].

CDC, 2025f. *Past Seasons’ Vaccine Effectiveness Estimates*. [online] Flu Vaccines Work. Available at: https://www.cdc.gov/flu-vaccines-work/php/effectiveness-studies/past-seasons-estimates.html [Accessed 21 Nov. 2025].

CDC, 2025g. *Pneumococcal Vaccination*. [online] Pneumococcal Disease. Available at: https://www.cdc.gov/pneumococcal/vaccines/index.html [Accessed 10 Mar. 2025].

CDC, 2025h. *RSV Vaccine Guidance for Adults*. [online] Respiratory Syncytial Virus Infection (RSV). Available at: https://www.cdc.gov/rsv/hcp/vaccine-clinical-guidance/adults.html [Accessed 24 Oct. 2025].

CDC, 2025i. *Staying Up to Date with COVID-19 Vaccines*. [online] COVID-19. Available at: https://www.cdc.gov/covid/vaccines/stay-up-to-date.html [Accessed 24 Oct. 2025].

CDC, 2025j. *Vaccination Coverage among Adults in the United States, National Health Interview Survey, 2022*. [online] AdultVaxView. Available at: https://www.cdc.gov/adultvaxview/publications-resources/adult-vaccination-coverage-2022.html [Accessed 29 Aug. 2025].

CMC, 2025. *Search the Physician Fee Schedule*. [online] Available at: https://www.cms.gov/medicare/physician-fee-schedule/search?Y=0&T=4&HT=0&CT=3&H1=90471&M=5 [Accessed 24 Oct. 2025].

Gourzoulidis, G., Barmpouni, M., Kossyvaki, V., Vietri, J. and Tzanetakos, C., 2023. Health and economic outcomes of 20-valent pneumococcal conjugate vaccine compared to 15-valent pneumococcal conjugate vaccine strategies for adults in Greece. *Frontiers in Public Health*, 11, p.1229524. 10.3389/fpubh.2023.1229524.

Grant, L.R., Meche, A., McGrath, L., Miles, A., Alfred, T., Yan, Q. and Chilson, E., 2023. Risk of Pneumococcal Disease in US Adults by Age and Risk Profile. *Open Forum Infectious Diseases*, 10(5), p.ofad192. 10.1093/ofid/ofad192.

Grosse, S.D., Krueger, K.V. and Pike, J., 2019. Estimated annual and lifetime labor productivity in the United States, 2016: implications for economic evaluations. *Journal of Medical Economics*, 22(6), pp.501–508. 10.1080/13696998.2018.1542520.

Herring, W.L., Zhang, Y., Shinde, V., Stoddard, J., Talbird, S.E. and Rosen, B., 2022. Clinical and economic outcomes associated with respiratory syncytial virus vaccination in older adults in the United States. *Vaccine*, 40(3), pp.483–493. 10.1016/j.vaccine.2021.12.002.

Hutton, D.W., Prosser, L.A., Rose, A.M., Mercon, K., Ortega-Sanchez, I.R., Leidner, A.J., Havers, F.P., Prill, M.M., Whitaker, M., Roper, L.E., Pike, J., Britton, A. and Melgar, M., 2024. Cost-effectiveness of vaccinating adults aged 60 years and older against respiratory syncytial virus. *Vaccine*, 42(24), p.126294. 10.1016/j.vaccine.2024.126294.

Jit, M., Brisson, M., Portnoy, A. and Hutubessy, R., 2014. Cost-effectiveness of female human papillomavirus vaccination in 179 countries: a PRIME modelling study. *The Lancet Global Health*, 2(7), pp.e406–e414. 10.1016/S2214-109X(14)70237-2.

Kearsley, A., 2025. *HHS Standard Values for Regulatory Analysis, 2025*. [online] Office of the Assistant Secretary for Planning and Evaluation, U.S. Department of Health and Human Services. Available at: https://aspe.hhs.gov/reports/standard-ria-values [Accessed 10 Mar. 2026].

Kim DeLuca, E., Gebremariam, A., Rose, A., Biggerstaff, M., Meltzer, M.I. and Prosser, L.A., 2023. Cost-effectiveness of routine annual influenza vaccination by age and risk status. *Vaccine*, 41(29), pp.4239–4248. 10.1016/j.vaccine.2023.04.069.

King, L.M., Andrejko, K.L., Kobayashi, M., Xing, W., Cohen, A.L., Self, W.H., Resser, J.J., Whitney, C.G., Baughman, A., Kio, M., Grijalva, C.G., Traenkner, J., Rouphael, N. and Lewnard, J.A., 2024. PNEUMOCOCCAL SEROTYPE DISTRIBUTION AND COVERAGE OF EXISTING AND PIPELINE PNEUMOCOCCAL VACCINES. *medRxiv*, p.2024.12.12.24318944. 10.1101/2024.12.12.24318944.

Kompaniyets, L., 2021. Underlying Medical Conditions and Severe Illness Among 540,667 Adults Hospitalized With COVID-19, March 2020–March 2021. *Preventing Chronic Disease*, [online] 18. 10.5888/pcd18.210123.

Kujawski, S.A., Whitaker, M., Ritchey, M.D., Reingold, A.L., Chai, S.J., Anderson, E.J., Openo, K.P., Monroe, M., Ryan, P., Bye, E., Como-Sabetti, K., Barney, G.R., Muse, A., Bennett, N.M., Felsen, C.B., Thomas, A., Crawford, C., Talbot, H.K., Schaffner, W., Gerber, S.I., Langley, G.E. and Kim, L., 2022. Rates of respiratory syncytial virus (RSV)-associated hospitalization among adults with congestive heart failure—United States, 2015–2017. *PLOS ONE*, 17(3), p.e0264890. 10.1371/journal.pone.0264890.

La, E.M., McGuiness, C.B., Singer, D., Yasuda, M. and Chen, C.-C., 2025. RSV vaccination uptake among adults aged 60 years and older in the United States during the 2023–2025 vaccination seasons. *Human Vaccines & Immunotherapeutics*, 21(1), p.2535755. 10.1080/21645515.2025.2535755.

Leidner, A.J. and Bletnitsky, S., 2024. *Summary of three economic analyses on the use of PCVs among 50-64 year old adults in the United States*.

Link-Gelles, P., Sean Chickery, Dhs., Alexander Webber, M.P.H., Toan C. Ong, P., Elizabeth A. K. Rowley, D., Malini B. DeSilva, M.D., Kristin Dascomb, M.D., Stephanie A. Irving, M.H.S., Nicola P. Klein, M.D., Shaun J. Grannis, M.D., Barron, M.A., Sarah E. Reese, P., Charlene McEvoy, M.D., Tamara Sheffield, M.D., Allison L. Naleway, P., Ousseny Zerbo, P., Colin Rogerson, M.D., Wesley H. Self, M.D., Yuwei Zhu, M.D., Adam S. Lauring, M.D., Emily T. Martin, P., Ithan D. Peltan, M.D., Adit A. Ginde, M.D., Nicholas M. Mohr, M.D., Kevin W. Gibbs, M.D., David N. Hager, M.D., Matthew E. Prekker, M.D., Amira Mohamed, M.D., Nicholas Johnson, M.D., Jay S. Steingrub, M.D., Akram Khan, M., Jamie R. Felzer, M.D., Abhijit Duggal, M.D., Jennifer G. Wilson, M.D., Nida Qadir, M.D., Christopher Mallow, M.D., Jennie H. Kwon, D.O., Cristie Columbus, M.D., Ivana A. Vaughn, P., Basmah Safdar, M.D., Jarrod M. Mosier, M.D., Estelle S. Harris, M.D., James D. Chappell, M.D., Natasha Halasa, M.D., Cassandra Johnson, M.S., Karthik Natarajan, P., Nathaniel M. Lewis, P., Sascha Ellington, P., Emily L. Reeves, M.P.H., Jennifer DeCuir, M.D., Meredith McMorrow, M.D., Clinton R. Paden, P., Amanda B. Payne, P., Fatimah S. Dawood, M.D., Diya Surie, M.D. and Collaborators, C.C.-19 V.E., 2025. Interim Estimates of 2024–2025 COVID-19 Vaccine Effectiveness Among Adults Aged ≥18 Years — VISION and IVY Networks, September 2024–January 2025. *MMWR. Morbidity and Mortality Weekly Report*, [online] 74. 10.15585/mmwr.mm7406a1.

Marrie, T.J., Tyrrell, G.J., Majumdar, S.R. and Eurich, D.T., 2017. Invasive Pneumococcal Disease: Still Lots to Learn and a Need for Standardized Data Collection Instruments. *Canadian Respiratory Journal*, 2017(1), p.2397429. 10.1155/2017/2397429.

Mendes, D., Averin, A., Atwood, M., Sato, R., Vyse, A., Campling, J., Weycker, D., Slack, M., Ellsbury, G. and Mugwagwa, T., 2022. Cost-effectiveness of using a 20-valent pneumococcal conjugate vaccine to directly protect adults in England at elevated risk of pneumococcal disease. *Expert Review of Pharmacoeconomics & Outcomes Research*, 22(8), pp.1285–1295. 10.1080/14737167.2022.2134120.

Moghadas, S.M., Shoukat, A., Bawden, C.E., Langley, J.M., Singer, B.H., Fitzpatrick, M.C. and Galvani, A.P., 2024. Cost-effectiveness of Prefusion F Protein-based Vaccines Against Respiratory Syncytial Virus Disease for Older Adults in the United States. *Clinical Infectious Diseases*, 78(5), pp.1328–1335. 10.1093/cid/ciad658.

Oligbu, G., Collins, S., Djennad, A., Sheppard, C.L., Fry, N.K., Andrews, N.J., Borrow, R., Ramsay, M.E. and Ladhani, S.N., 2019. Effect of Pneumococcal Conjugate Vaccines on Pneumococcal Meningitis, England and Wales, July 1, 2000–June 30, 2016 - Volume 25, Number 9—September 2019 - Emerging Infectious Diseases journal - CDC. [online] 10.3201/eid2509.180747.

Onwuchekwa, C., Moreo, L.M., Menon, S., Machado, B., Curcio, D., Kalina, W., Atwell, J.E., Gessner, B.D., Siapka, M., Agarwal, N., Rubbrecht, M., Nair, H., Rozenbaum, M., Aponte-Torres, Z., Vroling, H. and Begier, E., 2023. Underascertainment of Respiratory Syncytial Virus Infection in Adults Due to Diagnostic Testing Limitations: A Systematic Literature Review and Meta-analysis. *The Journal of Infectious Diseases*, 228(2), pp.173–184. 10.1093/infdis/jiad012.

Our World in Data, 2024. *COVID-19 vaccine doses administered by manufacturer*. [online] Our World in Data. Available at: https://ourworldindata.org/grapher/covid-vaccine-doses-by-manufacturer?time=2022-09-02..2024-03-31&country=~USA [Accessed 21 Nov. 2025].

Owusu-Edusei, K., Deb, A. and Johnson, K.D., 2022. Estimates of the Health and Economic Burden of Pneumococcal Infections Attributable to the 15-Valent Pneumococcal Conjugate Vaccine Serotypes in the USA. *Infectious Diseases and Therapy*, 11(3), pp.987–999. 10.1007/s40121-022-00588-x.

Patterson, S., Webber, C., Patton, M., Drews, W., Huijts, S.M., Bolkenbaas, M., Gruber, W.C., Scott, D.A. and Bonten, M.J.M., 2016. A post hoc assessment of duration of protection in CAPiTA (Community Acquired Pneumonia immunization Trial in Adults). *Trials in Vaccinology*, 5, pp.92–96. 10.1016/j.trivac.2016.04.004.

Prosser, L.A., Wallace, M., Rose, A.M., Mercon, K., Janusz, C.B., Gebremariam, A., Hutton, D.W., Leidner, A.J., Zhou, F., Ortega-Sanchez, I.R., Moulia, D., Link-Gelles, R., Saydah, S., Shah, M. and Pike, J., 2025. Cost-Effectiveness of 2023-2024 COVID-19 Vaccination in US Adults. *JAMA Network Open*, 8(8), p.e2523688. 10.1001/jamanetworkopen.2025.23688.

Ramirez, J., Carrico, R., Wilde, A., Junkins, A., Furmanek, S., Chandler, T., Schulz, P., Hubler, R., Peyrani, P., Liu, Q., Trivedi, S., Uppal, S., Kalina, W.V., Falsey, A.R., Walsh, E.E., Yacisin, K., Jodar, L., Gessner, B.D. and Begier, E., 2023. Diagnosis of Respiratory Syncytial Virus in Adults Substantially Increases When Adding Sputum, Saliva, and Serology Testing to Nasopharyngeal Swab RT–PCR. *Infectious Diseases and Therapy*, 12(6), pp.1593–1603. 10.1007/s40121-023-00805-1.

Reed, C., Chaves, S.S., Kirley, P.D., Emerson, R., Aragon, D., Hancock, E.B., Butler, L., Baumbach, J., Hollick, G., Bennett, N.M., Laidler, M.R., Thomas, A., Meltzer, M.I. and Finelli, L., 2015. Estimating Influenza Disease Burden from Population-Based Surveillance Data in the United States. *PLOS ONE*, 10(3), p.e0118369. 10.1371/journal.pone.0118369.

Robinson, L.A., Hammitt, J.K., Cecchini, M., Chalkidou, K., Claxton, K., Cropper, M.L., Eozenou, P., de Ferranti, D., Deolalikar, A.B., Campos Guanais de Aguiar, F., Jamison, D.T., Kwon, S., Lauer, J.A., O’Keeffe, L., Walker, D., Whittington, D., Wilkinson, T., Wilson, D. and Wong, B., 2019. Reference Case Guidelines for Benefit-Cost Analysis in Global Health and Development. *SSRN Electronic Journal*. [online] 10.2139/ssrn.4015886.

Sah, P., Fitzpatrick, M.C., Zimmer, C.F., Abdollahi, E., Juden-Kelly, L., Moghadas, S.M., Singer, B.H. and Galvani, A.P., 2021. *Asymptomatic SARS-CoV-2 infection: A systematic review and meta-analysis | PNAS*. [online] Available at: https://www.pnas.org/doi/abs/10.1073/pnas.2109229118?url_ver=Z39.88-2003&rfr_id=ori%3Arid%3Acrossref.org&rfr_dat=cr_pub++0pubmed [Accessed 21 Nov. 2025].

Stoecker, C., 2024. Economic Analysis and Public Health Impact of PCV.

Suaya, J.A., Jiang, Q., Scott, D.A., Gruber, W.C., Webber, C., Schmoele-Thoma, B., Hall-Murray, C.K., Jodar, L. and Isturiz, R.E., 2018. Post hoc analysis of the efficacy of the 13-valent pneumococcal conjugate vaccine against vaccine-type community-acquired pneumonia in at-risk older adults. *Vaccine*, 36(11), pp.1477–1483. 10.1016/j.vaccine.2018.01.049.

Talbird, S.E., La, E.M., Carrico, J., Poston, S., Poirrier, J.-E., DeMartino, J.K. and Hogea, C.S., 2021. Impact of population aging on the burden of vaccine-preventable diseases among older adults in the United States. *Human Vaccines & Immunotherapeutics*, 17(2), pp.332–343. 10.1080/21645515.2020.1780847.

Talbird, S.E., Poston, S. and Hogea, C.S., 2017. *A BUDGET-IMPACT ANALYSIS OF QUADRIVALENT INFLUENZA VACCINE USE IN THE UNITED STATES*. [online] Available at: https://www.ispor.org/heor-resources/presentations-database/presentation/ispor-22nd-annual-international-meeting/a-budget-impact-analysis-of-quadrivalent-influenza-vaccine-use-in-the-united-states [Accessed 24 Oct. 2025].

Tartof, S.Y., Aliabadi, N., Goodwin, G., Slezak, J., Hong, V., Ackerson, B., Ackerson, B., Liu, Q., Shaw, S., Welsh, S., Stern, J., Kapadia, B., Spence, B., Lewnard, J., Davis, G., Aragones, M., Dutro, M., Chilson, E., Gonzalez, E., Hubler, R., Miller, A., Chia, B., Jodar, L., Gessner, B.D. and Begier, E., 2025a. 165. Real-world Abrysvo Vaccine Effectiveness (VE) against Respiratory Syncytial Virus (RSV)-related Severe Acute Respiratory Infection (ARI) Hospitalizations and Emergency Department (ED) Visits—Kaiser Permanente of Southern California (KPSC), November 2023–April 2024. *Open Forum Infectious Diseases*, 12(Supplement_1), p.ofae631.002. 10.1093/ofid/ofae631.002.

Tartof, S.Y., Aliabadi, N., Goodwin, G., Slezak, J., Hong, V., Ackerson, B., Liu, Q., Shaw, S., Welsh, S., Kapadia, B., Spence, B.C., Lewnard, J.A., Davis, G.S., Aragones, M., Dutro, M., Chilson, E., Gonzalez, E., Hubler, R., Jodar, L., Gessner, B.D. and Begier, E., 2025b. Estimated Vaccine Effectiveness for Respiratory Syncytial Virus-Related Acute Respiratory Illness in Older Adults: Findings From the First Postlicensure Season. *Clinical Infectious Diseases: An Official Publication of the Infectious Diseases Society of America*, p.ciaf496. 10.1093/cid/ciaf496.

The White House, 2023. *OMB Circular A-94, Appendix D*. Available at: https://www.whitehouse.gov/wp-content/uploads/2023/11/CircularA-94AppendixD.pdf [Accessed 12 Aug. 2025].

United Nations, Department of Economic and Social Affairs, Population Division, 2024. *World Population Prospects 2024*. [online] Available at: https://population.un.org/wpp/downloads?folder=Standard%20Projections&group=Most%20used [Accessed 23 Oct. 2025].

U.S. Bureau of Labor Statistics, 2025a. *Civilian labor force participation rate by age, sex, race, and ethnicity*. [online] Bureau of Labor Statistics. Available at: https://www.bls.gov/emp/tables/civilian-labor-force-participation-rate.htm [Accessed 10 Mar. 2026].

U.S. Bureau of Labor Statistics, 2025b. *E-16. Unemployment rates by age, sex, race, and Hispanic or Latino ethnicity*. [online] Bureau of Labor Statistics. Available at: https://www.bls.gov/web/empsit/cpsee_e16.htm [Accessed 10 Mar. 2026].

US Census Bureau, 2025. *National Population by Characteristics: 2020-2024*. [online] Census.gov. Available at: https://www.census.gov/data/tables/time-series/demo/popest/2020s-national-detail.html [Accessed 21 Nov. 2025].

Walsh, E.E., Pérez Marc, G., Zareba, A.M., Falsey, A.R., Jiang, Q., Patton, M., Polack, F.P., Llapur, C., Doreski, P.A., Ilangovan, K., Rämet, M., Fukushima, Y., Hussen, N., Bont, L.J., Cardona, J., DeHaan, E., Castillo Villa, G., Ingilizova, M., Eiras, D., Mikati, T., Shah, R.N., Schneider, K., Cooper, D., Koury, K., Lino, M.-M., Anderson, A.S., Jansen, K.U., Swanson, K.A., Gurtman, A., Gruber, W.C., Schmoele-Thoma, B., and RENOIR Clinical Trial Group, 2023. Efficacy and Safety of a Bivalent RSV Prefusion F Vaccine in Older Adults. *The New England Journal of Medicine*, 388(16), pp.1465–1477. 10.1056/NEJMoa2213836.

Wateska, A.R., Nowalk, M.P., Lin, C.J., Harrison, L.H., Schaffner, W., Zimmerman, R.K. and Smith, K.J., 2020. Cost-Effectiveness of Pneumococcal Vaccination and Uptake Improvement Programs in Underserved and General Population Adults Aged < 65 years. *Journal of community health*, 45(1), pp.111–120. 10.1007/s10900-019-00716-8.

van Werkhoven, C.H., Huijts, S.M., Bolkenbaas, M., Grobbee, D.E. and Bonten, M.J.M., 2015. The Impact of Age on the Efficacy of 13-valent Pneumococcal Conjugate Vaccine in Elderly. *Clinical Infectious Diseases: An Official Publication of the Infectious Diseases Society of America*, 61(12), pp.1835–1838. 10.1093/cid/civ686.

Wisk, L.E. and Sharma, N., 2025. *Prevalence and Trends in Pediatric-Onset Chronic Conditions in the United States, 1999–2018 - Academic Pediatrics*. [online] Available at: https://www.academicpedsjnl.net/article/S1876-2859(25)00035-X/fulltext [Accessed 27 Oct. 2025].

Yehoshua, A., Cook, A.D., Di Fusco, M., Rudolph, A.E., Thoburn, E., Lopez, S.M.C., Yarnoff, B. and Andersen, K.M., 2024. Health outcomes and economic burden among patients with a COVID-19-associated hospitalization in the United States during the predominance of the XBB and JN.1 omicron lineages. *Journal of Medical Economics*, 27(1), pp.1372–1378. 10.1080/13696998.2024.2416873.
